# *scpp5* regulates tooth development and injury-induced repair in zebrafish through mineralization and Wnt/β-catenin signaling

**DOI:** 10.1016/j.mocell.2026.100377

**Published:** 2026-06-03

**Authors:** Qiqi Liu, Weifeng Hao, Zhenan Zhang, Yu Yue, Deqin Yang

**Affiliations:** 1 Department of Endodontics, The Affiliated Stomatological Hospital of Chongqing Medical University, Chongqing 404100, China; 2 Chongqing Key Laboratory of Oral Diseases, Chongqing 404100, China; 3 Chongqing Municipal Key Laboratory of Oral Biomedical Engineering of Higher Education, Chongqing 404100, China; 4 Chongqing Municipal Health Commission Key Laboratory of Oral Biomedical Engineering, Chongqing 404100, China; 5 Department of Conservative Dentistry and Endodontics, Shanghai Stomatological Hospital & School of Stomatology, Fudan University, Shanghai 200001, China; 6 Shanghai Key Laboratory of Craniomaxillofacial Development and Diseases, Fudan University, Shanghai 200001, China

**Keywords:** SCPP5, Tooth development, Tooth mineralization, Tooth repair, Wnt/β-catenin

## Abstract

A member of the secretory calcium-binding phosphoprotein (SCPP) family, scpp5 is specifically expressed in zebrafish tooth germ. Although preliminary data indicates that scpp5 deficiency impairs tooth mineralization, its molecular mechanism and role in tooth repair remain unclear. In this study, we generated *scpp5*^*-/-*^ knockout and *Tg(hsp70l:scpp5-GFP; cryaa:venus)* overexpression zebrafish lines. Tooth germ cells were labeled using *Tg(dlx2b:Dendra2-NTR)*, and a tooth injury model was established via the nitroreductase (NTR)/metronidazole (MTZ) system. Loss-of-function studies showed that *scpp5* knockout inhibited tooth mineralization and tooth germ cell development during normal development, attenuated Wnt/β-catenin signaling, downregulated calcium efflux channel gene expression, and suppressed both enameloid and dentin matrix-related genes. Rescue of Wnt pathway activity restored calcium efflux channel and dentin matrix gene expression, but not enameloid matrix gene expression. Gain-of-function studies revealed that *scpp5* overexpression did not affect normal tooth development, but during repair after injury, it accelerated mineralization and cell regeneration. Mechanistically, *scpp5* overexpression during repair activated Wnt/β-catenin signaling, specifically enhancing calcium channel and dentin matrix gene expression, without affecting enameloid matrix gene expression. Collectively, these findings demonstrate that scpp5 promotes tooth mineralization during normal development via Wnt/β-catenin-dependent regulation of both enameloid and dentin matrix genes, whereas during injury repair, scpp5 facilitates regeneration by selectively activating Wnt/β-catenin to drive dentin matrix and calcium channel gene expression, independent of enameloid matrix genes.

## Graphical Abstract

**Figure:**
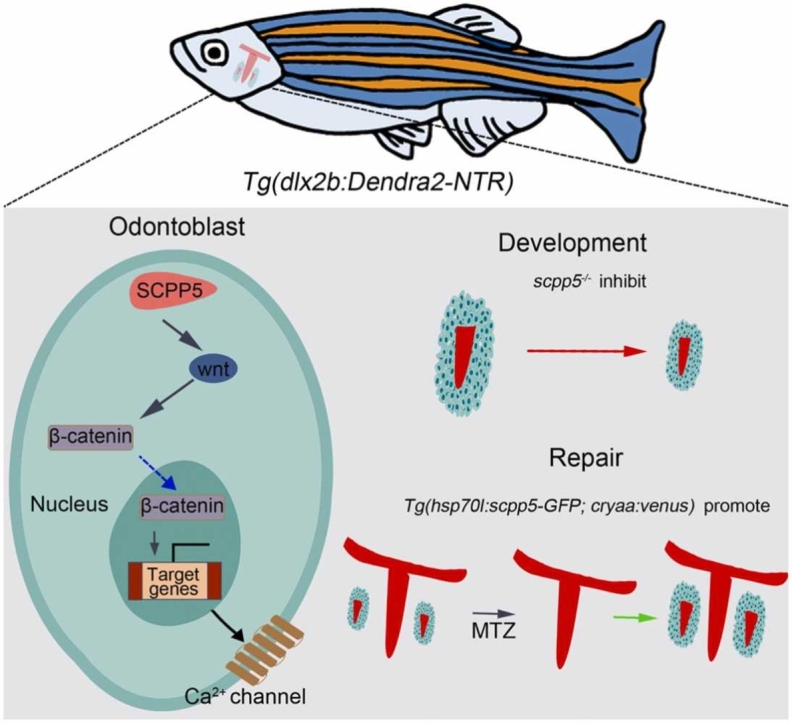

## INTRODUCTION

The formation of highly mineralized tooth hard tissues initiates with the organized deposition of hydroxyapatite crystals within the extracellular organic matrix. Subsequently, intracellular calcium (Ca²⁺) facilitates the growth and maturation of these hydroxyapatite crystals (Shapiro et al., 2025). To investigate the molecular mechanisms underlying tooth mineralization during development and repair following injury, numerous studies have leveraged the continuously growing incisors of rodents as an experimental model (Kegulian et al., 2024). However, the lifelong tooth regenerative capacity of zebrafish provides distinct advantages for studying tooth mineralization mechanisms.

Members of the secretory calcium-binding phosphoproteins (SCPP) family have been identified as critical regulators of calcium metabolism in multiple mineralized tissues, mediating mineralization processes in mammalian teeth and bones (Kawasaki, 2011, Lv et al., 2017). During tooth development and mineralization, proteins of the SCPP family are primarily secreted by 3 key cell types: ameloblasts, inner dental epithelial (IDE) cells, and odontoblasts. These secreted matrix proteins play essential roles in the formation and mineralization of enamel, enameloid, and dentin matrices (Kawasaki et al., 2021). Clinically, deficient SCPP synthesis or secretion in humans manifests as disrupted enamel and dentin mineralization, resulting in developmental tooth disorders including dentin dysplasia, dentinogenesis imperfecta, and amelogenesis imperfecta, which ultimately impair masticatory function and quality of life (Kawasaki and Weiss, 2008).

The SCPP family comprises 2 functionally distinct subgroups, based on their structural and mineralization properties: (1) acidic SCPPs that regulate bone and dentin mineralization, and (2) P/Q-rich SCPPs that mediate enamel and enameloid formation (Leurs et al., 2022). As a P/Q-rich SCPP family member, *scpp5* demonstrates specialized expression restricted to tooth tissue in teleost fishes (Kawasaki et al., 2021). Current research demonstrates that the cell-type-specific expression profile of *scpp5*, restricted to dental epithelial and mesenchymal lineages but absent in osteogenic cells, provides a molecular marker for distinguishing tooth-forming from bone-forming cell populations (Kawasaki, 2009, Rosa et al., 2021). Recent studies have revealed differential expression patterns of *scpp5* in different species, with significantly higher expression levels observed in species with larger pharyngeal teeth (Karagic et al., 2020). Moreover, *scpp5* emerges as the highest expression levels among all SCPP family genes during tooth development in the tropical gar. This striking spatial expression gradient has generated substantial research interest in understanding the precise role of *scpp5* in tooth development and mineralization processes (Karagic et al., 2020).

In the adult zebrafish, teeth are arranged in 3 rows that run rostro-caudally: the ventral (5 teeth), mediodorsal (4 teeth), and dorsal (2 teeth) rows (Van der Heyden and Huysseune, 2000). Zebrafish tooth development occurs through 5 distinct yet temporally overlapping phases: initiation, morphogenesis, early cytodifferentiation, late cytodifferentiation, and attachment (Verstraeten et al., 2016). The development of the first tooth is initiated at 48 h post-fertilization (hpf) at position 4 in the ventral row. This tooth is designated 4V1, where “1” denotes the first generation (Yu et al., 2015). Transcriptional activation of *scpp5* was first identified in the late cytodifferentiation phase of odontogenesis in 4V1 (Rosa et al., 2021). To gain deeper insights into the role of SCPP5 in tooth development and mineralization, a *scpp5* knockout zebrafish line was created. The results showed that 45.8% of *scpp5*^*-/-*^ zebrafish developed fewer functional teeth. Researchers postulated that this defect might be attributed to delayed or blocked tooth replacement, though the precise mechanism remains unclear (Qu et al., 2021).

It should be noted that, although scpp5 is non-functional or absent in mammals (Kawasaki et al., 2021), the downstream tooth mineralization mechanisms that it engages in zebrafish may be evolutionarily conserved. In this study, *scpp5*^*-/-*^ and *Tg(hsp70l:scpp5-GFP; cryaa:venus)* zebrafish were established to investigate the effects of *scpp5* loss and overexpression on mineralization in zebrafish tooth development. Subsequently, the *Tg(dlx2b:Dendra2-NTR)* were utilized to label zebrafish tooth germ cells and establish a tooth injury model by the nitroreductase (NTR)/metronidazole (MTZ) system (Liu et al., 2023). More importantly, the involvement of the Wnt/β-catenin signaling pathway in SCPP5-mediated regulation of mineralization, during both tooth development and repair following injury in zebrafish were identified and validated. This discovery provides novel insights into the role of SCPP5 in regulating tooth development and repair following injury.

## MATERIALS AND METHODS

### Animals

Zebrafish of the AB genetic background were used as wild-type (WT) zebrafish. The environmental conditions of the zebrafish fish room were standard laboratory conditions: temperature 28.5°C; photoperiod 14-h light cycle/10-h dark cycle. The use of animals and all animal procedures were approved by the Ethics Committee of the Stomatological Hospital of Chongqing Medical University (Approval No.2022163). All experiments were performed following the guidelines of ARRIVE (Animal Research: Reporting of In Vivo Experiments).

### Generation of Plasmids and Transgenic Lines

To construct the *pBluescript-scpp5:Dendra2-NTR* plasmid, the 4 900-bp promoter of *scpp5* was cloned from 3 days post-fertilization (dpf) zebrafish genomics DNA, and the following primer sequences were used for PCR amplification: forward: 5′-AGGGGGCCCCCTCGAAGATCGAGACCGTAGCA-3′ and reverse: 5′-CCCACCGGTGCCGGAGAGACGTCCACATGGTT-3′. These promoter sequences were sub-cloned into *pBluescript-Dendra2-NTR* vector between the *Apa*I (NEB) and *Age*I (NEB) enzyme sites. The hsp70l is a heat-shock promoter that drives gene expression after 30-40 min of heat shock at 38.5°C (Shen et al., 2013, Shoji and Sato-Maeda, 2008). To generate the *pBluescript-hsp70l:scpp5-GFP; cryaa:venus* plasmid and *pBluescript-hsp70l:wnt10a-GFP; cryaa:venus* plasmid, zebrafish *scpp5* and *wnt10a* full-length coding sequences was cloned from 3 dpf cDNA. The following primer sequences were used for PCR amplification: *scpp5* forward: 5′-GTCGACATGTGGACGTCTCTCCTGTGT-3′ *scpp5* reverse: 5′-CTCGAGATCTGAGTCCGGATGGTCTGGTAGACACCTGTCC-3′. *Wnt10a* forward: 5′-GTCGACATGAGCTCTCACGACATCAG-3′ and *wnt10a* reverse: 5′-CTCGAGATCTGAGTCCGGATTTGCAGACACTGACCCACT-3′. These sequences were sub-cloned into *pBluescript-hsp70l:GFP; cryaa:venus* plasmid vector between the *Sal*I (NEB) and *Xho*I (NEB) enzyme sites. The *cryaa:venus* cassette served as a visual marker for successful transgenesis, with Venus fluorescence in the lens confirming the presence of the entire transgenic construct, including the hsp70l promoter-driven element (Zhong et al., 2018). The constructs *pBluescript-hsp70l:scpp5-GFP; cryaa:venus*, *pBluescript-hsp70l:wnt10a-GFP; cryaa:venus,* and *pBluescript-scpp5:Dendra2-NTR* were co-injected with I-SceI (NEB) into the one-cell stage of embryos under the AB genetic background for transgenesis. All transgenic lines were outcrossed at least every other generation to ensure genetic diversity. *Tg(dlx2b:Dendra2-NTR)* was generated as previously reported (Liu et al., 2023).

### Generated *scpp5* Mutants Using CRISPR/Cas9 System

*Scpp5* mutants were generated by targeting the 4th exon of *scpp5* with CRISPR/Cas9 technology. The genomic region flanking the gRNA target site was amplified with *scpp5*-specific primers: forward: 5′-TAATACGACTCACTATAGGAGGAGCAGCGGGAACATGTTTTAGAGCTAGAAATAGC-3′ and reverse: 5′- AAAAAAAGCACCGACTCGGT-3′. The PCR product was used to synthesize gRNA with T7 RNA Polymerase (NEB). The Cas9 mRNA (300 ng/µl) and gRNA (100-400 ng/µl) were co-injected into one-cell stage of embryos under the AB genetic background. The embryos were raised to adulthood and then outcrossed with WT fish to select founders carrying mutations. Mutations were further confirmed via DNA sequencing. The validating sequence was amplified by the following primers: F: 5′-GCTCGTCATTTTCCAGCCTG-3′ R: 5′-TGTGTGGGGAATGACTGAGG-3′. A mutant allele with the targeted 2-bp deletion was identified and subsequently outcrossed to the wild type.

To compare the SCPP5 protein expression level in WT and mutant, the WT *scpp5* coding sequences were amplified from 3 dpf WT cDNA using the following primers: forward: 5′-GTCGACCGGTATGTGGACGTCTCTCCTGTGT-3′ and reverse: 5′-ACTAGTTGGTCTGGTAGACACCTGTCC-3′. The mutant *scpp5* coding sequence was constructed by overlap extension PCR using 3 dpf WT cDNA as the template and the following primers: First primer forward: 5′-GTCGACCGGTATGTGGACGTCTCTCCTGTGTCTT-3′ First primer reverse: 5′-GCTGGATTAGCAGGGAATCGGAGGGAAAATGATTTCCATGC-3′. Second primer forward: 5′-TGGAAATCATTTTCCCTCCGATTCCCTGCTAATCCAGCAGG-3′ and Second primer reverse: 5′-ACTAGTTGGTCTGGTAGACACCTGT-3′. The WT and mutant *scpp5* coding sequences were inserted between the *Sal*I (NEB) and *Spe*I (NEB) enzyme sites in the *pBluescript-hsp70l:p2a-DsRed; cryaa:venus* plasmid. The *pBluescript-hsp70l: scpp5*^*+/+*^*-p2a-DsRed; cryaa:venus* plasmid, and *pBluescript-hsp70l: scpp5*^*-/-*^*-p2a-DsRed; cryaa:venus* plasmid were co-injected with I-SceI (NEB) into the one-cell stage of embryos under the AB genetic background.

### Drug Treatment

MTZ (MCE) was dissolved in egg water with 0.003% PTU and 0.2% DMSO (Sangon Biotech), and its final concentration was 12 mM. The standard egg water contained ≤1 mM calcium (Ca) and ≤1 mM phosphorus (P). To create a high Ca environment, the standard egg water was supplemented with CaCl₂, adjusting the Ca concentration to 30 mM. A high-phosphate environment was created by supplementing standard egg water with compound sodium phosphate (CSP; a 1:1 molar mixture of Na_2_HPO_4_ and NaH_2_PO_4_) to a final phosphorus concentration of 30 mM. For small molecule treatment, the larvae were incubated egg water with SKL2001 (20 µM, MCE) and Zamaporvint (100 µM, MCE) to activate and inhibit Wnt-β-catenin signaling, respectively. The chemical solutions were replenished at 24-h intervals to ensure sustained pharmacological activity.

### Micro-CT Analysis

Zebrafish were fixed with 4% paraformaldehyde (PFA) overnight at 4°C. Micro-CT scanning was performed using a SkyScan 1276 system (Bruker, Kontich, Belgium) with the following parameters: X-ray source voltage 80 kV, current 80 μA, exposure time 300 ms, rotation step 0.4°, frame averaging of 3, and 360° scanning. The isotropic voxel size was 6 μm. Raw projection images were reconstructed into cross-sectional slices using NRecon software (version 1.7.4.6, Bruker) with dynamic image range correction, beam hardening correction of 25%, and a smoothing kernel of 2.

### Scanning Electron Microscope and Energy Spectrum Analysis

Zebrafish teeth were carefully dissected, rinsed twice in double-distilled water, and freeze-dried for 4 h. Dried samples were mounted on aluminum stubs and sputter-coated with a 5 nm palladium-gold film (Leica EM ACE600). SEM imaging was performed using a Gemini 300 (Zeiss, Oberkochen, Germany) at an accelerating voltage of 5 kV, working distance of 8 mm, and probe current of 100 pA. Images were acquired at magnifications ranging from 500× to 10,000×.

For elemental analysis, energy-dispersive X-ray spectroscopy was conducted using an Xplore detector (Oxford Instruments, Abingdon, UK) at 15 kV accelerating voltage, working distance of 8.5 mm, and acquisition time of 60 s (live time). Spectra were processed using AZtec software. The analysis region (ROI) was defined as the entire tooth crown surface. Quantitative data (weight % and atomic % of calcium, phosphorus, oxygen, carbon, and other detectable elements) were calculated using the ZAF correction method. At least 3 teeth per group and 3 measurement spots per tooth were analyzed to obtain the mean ± standard deviation.

### Alizarin Red Staining

The larvae fixed by 4% FPA were washed twice with PBST. The ventral skin, yolk, and heart were carefully removed. Following a 30-min fixation in 50% ethanol at room temperature (RT), larvae were stained with 0.05% alizarin red solution (Sangon Biotech) in the dark. Next, the bleach solution (1.5% H_2_O_2_ and 1% KOH) was left uncapped at RT for 20 min. Finally, larvae were treated with a solution of 20% glycerol and 25% KOH, followed by continuous oscillation at RT for 10 h. Pictures were taken under confocal laser microscopy (LSM780, Carl Zeiss).

### Whole Mount In Situ Hybridization

The fixed larvae were rehydrated through a graded PBST series (25%, 50%, 75%, 100%) before digesting with Proteinase K (PK) for 30 min at RT. In order to terminate PK activity, larvae were post-fixed in 4% PFA for 30 min at RT. Prior to probe addition, larvae were pre-hybridized for 3 h at 68.5°C and then hybridized overnight in a 68.5°C water bath with prepared probes (Table S1). 25%, 50%, 75%, and 100% SSCT solutions were used for gradient washing after removing the probe at 68.5°C. A graded series of MABT (25%, 50%, 75%, 100%) was applied for gradual buffer exchange SSCT at RT. Primary antibody labeling was performed using Anti-Dig-AP (1:2 000 dilution) (Roche) in blocking buffer at 4°C for 12 to 16 h after incubated with 1X blocking at RT for 3 to 5 h. The antibody solution was aspirated, followed by 8 washes with MABT buffer (15 min each). The larvae were incubated with BCIP/NBT solution at 37°C under light-protected conditions until the desired signal developed. The staining reaction was quenched with a stop solution (0.05 μM phosphate buffer, 1 mM EDTA, 0.1% Tween-20) at RT and captured under a microscope (Leiss, SteREO DiscoveryV20).

### Antibody Staining

After ventral skin, yolk, and heart were carefully removed, 8 times of PT washing (15 min each) and 1 h blocking at 4°C was performed using PBTN. The primary antibodies—including anti-Dendra2 (1:1,000, TA180094, Origene), anti-mCherry (1:1,000, AB0040, Origene), and anti-β-catenin (1:1,000, AB227499, Abcam)—were incubated overnight at 4°C. After 8 15-min washes with PT buffer and a 1-hour block with PBTN at 4°C, larvae were incubated overnight at 4°C in the dark with the following secondary antibodies: Alexa Fluor 488 (1:1,000, A-21202, Invitrogen) and Alexa Fluor 568 (1:1,000, A-10042, Invitrogen). Following an additional 8 15-min PT washes, the stained larvae were imaged using a confocal laser scanning microscope (LSM780, Carl Zeiss).

### Fluorescence In Situ Hybridization (FISH)

To remove endogenous peroxidase, the fixed larvae were incubated in bleaching solution (3% H₂O₂ in methanol) for at least 1 h at RT with gentle agitation. Following thorough bleaching, larvae were rehydrated through a graded PBST series (25%, 50%, 75%, 100%) before removing ventral skin, yolk, and heart. The fixed larvae were pre-hybridized for 3 h at 65°C prior to probe addition and then hybridized overnight in a 65°C water bath with prepared probes (Table S1). The Anti Dig-POD (Roche) was incubated at 4°C overnight, following PT wash 8 times (15 min each) and applied overnight by Cy3-conjugated tyramide signal amplification (Roche) at RT. The following steps were the same as those for antibody staining.

### Reverse Transcription Quantitative Polymerase Chain Reaction

Following euthanasia, larvae were micro-dissected to isolate the fifth pharyngeal arch, with all other tissues removed. The total RNA was extracted using the NucleoZOL (MACHEREY-NAGEL), and total RNA was then reverse-transcribed into cDNA using the Omniscript-Reverse Transcriptase Kit (QIAGEN). The FastStart Universal SYBR Green Master (Roche) was used for the real-time quantitative PCR reaction. The relative expression levels were calculated using *gapdh* as the housekeeping gene. The primer sequences are shown in Table S2.

### Heat-Shock Treatment

For the heat-shock experiments, *Tg(hsp70l: scpp5*^*+/+*^*-p2a-DsRed; cryaa:venus), Tg(hsp70l: scpp5*^*-/-*^*-p2a-DsRed; cryaa:venus), Tg(hsp70l:scpp5-GFP; cryaa:venus),* and *Tg(hsp70l:wnt10a-GFP; cryaa:venus)* larvae were transferred into 60 mm petri dishes containing 10 mL of system water and placed in a precision water bath preset at 38.5°C. The water temperature inside the petri dishes was verified using a calibrated digital thermometer (accuracy ±0.1°C). Heat-shock was performed at 38.5°C for 40 min, after which the larvae were immediately returned to fresh system water at 28.5°C for recovery. The treatment was repeated every 12 h for the desired period. Control larvae of the same transgenic lines were handled identically but maintained at 28.5°C throughout without heat-shock. For each transgenic line, approximately 30 larvae per group were subjected to heat-shock.

### Statistical Analysis

The results were statistically analyzed using GraphPad Prism 9 (GraphPad Software). For all experiments, a biological replicate was defined as an individual zebrafish larva or adult fish, each derived from an independent cross. Technical replicates (eg, repeated measurements from the same individual) were not used for statistical analysis, except for RT-qPCR, where each sample was measured in triplicate, and the average value was used for subsequent biological replicate analysis. Student's *t* test was employed for comparisons between 2 independent groups. Comparisons across multiple independent groups were analyzed by one-way ANOVA. Tukey was subsequently applied for multiple comparisons in one-way ANOVA models. All quantitative data are presented as mean ± standard deviation (SD) from at least 3 biologically independent zebrafish per group, unless otherwise specified in the figure legends. A value of *P* < .05 was considered to be statistically significant.

## RESULTS

### The Role of SCPP5 in Tooth Mineralization During Development

**Fig. 1:**
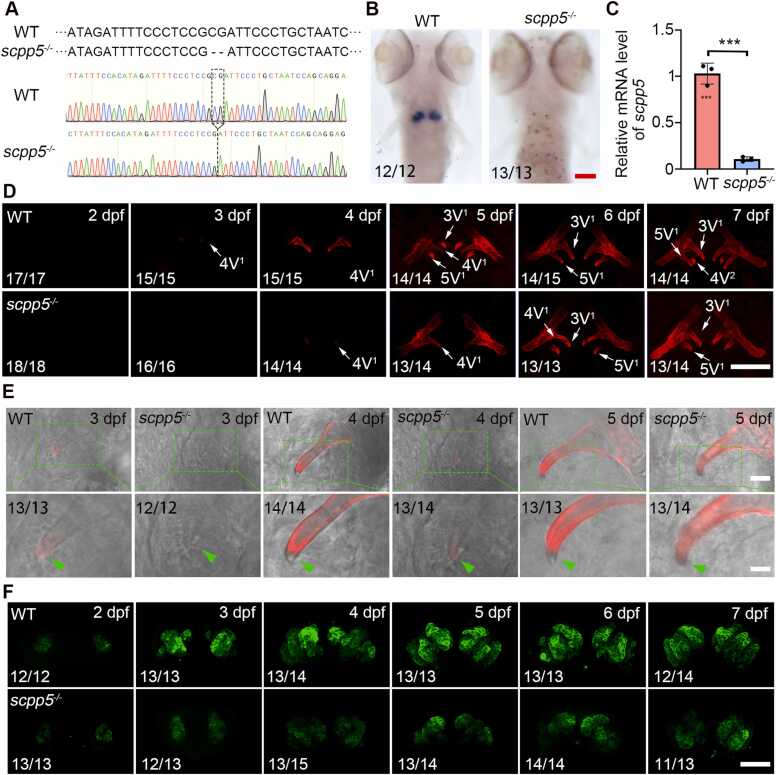
Knockout of *scpp5* impaired zebrafish tooth development. (A) The DNA base sequence map showed that *scpp5*^*-/-*^ zebrafish was missing 2 bases of CG relative to WT. (B) WISH showed the *scpp5* expression at 6 dpf (scale bars, 100 µm). (C) RT-qPCR showed the *scpp5* expression at 6 dpf. (D) Three-dimensional reconstruction from Z-stack images of zebrafish teeth under alizarin red staining from 2 to 7 dpf (scale bars, 100 µm). (E) Two-dimensional sectioned images of 4V1 under alizarin red staining and brightfield views from 3 to 5 dpf (scale bars, up 200 µm, down 100 µm). (F) Antibody staining of Dendra2 was performed to show the development of tooth germ cells from 2 to 7 dpf (scale bars, 50 µm). The bar graph presents the mean and standard deviation, *P* value *** *P* < .001 was calculated by *t*-test. 3V1, the first generation tooth at position 3 in the ventral row; dpf, day post-fertilization; WISH, whole mount in situ hybridization; WT, wild type; green arrowheads, 4V1 enameloid.

A loss-of-function mutant was generated for *scpp5* in zebrafish using CRISPR/Cas9, which introduced a 2-bp deletion at positions 190-191 relative to the initiation codon (Fig. 1A). Both WISH and RT-qPCR showed a marked decrease in the *scpp5* mRNA expression in the tooth tissues of the mutant (Fig. 1B and C). To further investigate the molecular consequence of this mutation, at first, attempts were made to amplify the SCPP5 coding sequence from 3 dpf mutant complementary DNA (cDNA) using the same primers as for the WT. However, no specific amplification product was detected from the mutant sample (Fig. S1A), suggesting that the mutation interferes with transcript stability. Therefore, the mutant *scpp5* coding sequence was constructed by overlap extension PCR based on the 3 dpf WT cDNA. The resulting WT and mutant *scpp5* coding sequences were then inserted into the *pBluescript-hsp70l:p2a-DsRed; cryaa:venus* plasmid, yielding *Tg(hsp70l: scpp5*^*+/+*^*-p2a-DsRed; cryaa:venus)* and *Tg(hsp70l: scpp5*^*-/-*^*-p2a-DsRed; cryaa:venus)*, respectively. Upon heat shock induction, *Tg(hsp70l: scpp5*^*+/+*^*-p2a-DsRed; cryaa:venus)* showed strong DsRed fluorescence, whereas *Tg(hsp70l: scpp5*^*-/-*^*-p2a-DsRed; cryaa:venus)* exhibited no detectable signal, confirming that the mutant coding sequence failed to produce a functional protein (Fig. S1B).

Despite the initiation of tooth mineralization in zebrafish at 2-3 dpf (Yu et al., 2015), a tooth mineralization absence was observed in approximately 20.59% (21/102) of *scpp5*^*-/-*^ zebrafish at 9 dpf (Fig. S1C and Table S3). Furthermore, even in *scpp5*^*-/-*^ zebrafish with normal tooth counts, dentin mineralization was consistently delayed (Fig. 1D). At 3 dpf, the 4V1 enameloid was alizarin red-positive in both WT and *scpp5*^*-/-*^ zebrafish (Fig. 1E). In WT zebrafish, the staining was no longer detectable at 4 dpf, a time point that coincided with the tooth's functional attachment to the fifth cerato-branchial and the completion of mineralization (Fig. 1E). In contrast, although the 4V1 tooth in *scpp5*^*-/-*^ zebrafish achieved functional attachment by 5 dpf, its enameloid remarkably retained alizarin red staining at this stage (Fig. 1E). Using the *Tg(dlx2b:Dendra2-NTR)* line established in the present study to label tooth germ cells (Liu et al., 2023), a significant developmental delay was observed in the tooth germ cells of *scpp5*^*-/-*^ zebrafish compared to WT from 3 to 7 dpf (Fig. 1F).

**Fig. 2:**
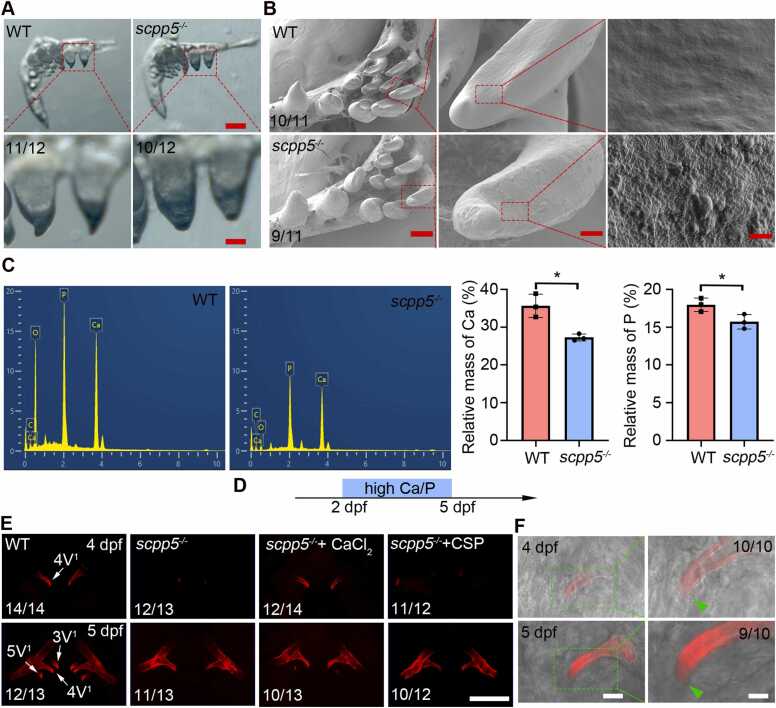
The tooth surfaces morphology of WT and *scpp5*^*-/-*^ adult zebrafish and the influence of high calcium and high phosphorus on the mineralization of *scpp5*^*-/-*^ zebrafish. (A) Microscopic observation of adult zebrafish teeth at 3 mpf (scale bars, uper 200 µm, down 50 µm F). (B) Scanning electron microscopy of adult zebrafish teeth at 3 mpf in different magnification images (scale bars, left-right 100 µm, 20 µm, 5 µm). (C) Energy dispersive spectrometer analysis in (B). (D) Experimental schedule. Zebrafish were treated with high calcium (30 mM) and high phosphorus (30 mM) from 2 to 5 dpf. (E) Three-dimensional reconstruction from Z-stack images of zebrafish teeth under alizarin red staining at 4 and 5 dpf (scale bars, 100 µm). (F) Two-dimensional sectioned images of 4V1 under alizarin red staining and brightfield views in the high calcium group from at 4 and 5 dpf (scale bars, left 200 µm, right 100 µm). The bar graph presents the mean and standard deviation, *P* value * *P* < .05 was calculated by *t*-test. 3V1, the first generation-tooth at position 3 in the ventral row; Ca, calcium; CSP, compound sodium phosphate (a 1:1 molar mixture of Na₂HPO₄ and NaH₂PO₄); dpf, days post-fertilization; mpf, months post-fertilization; P, phosphorus; WT, wild type; green arrowheads, 4V1 enameloid.

In adult zebrafish, *scpp5*^*-/-*^ zebrafish exhibited abnormal tooth cusp morphology (Fig. 2A), while micro-CT reconstruction showed no significant skeletal differences (Fig. S1D). SEM analysis revealed markedly rougher tooth surfaces in *scpp5*^*-/-*^ zebrafish, characterized by larger pit-like structures (Fig. 2B). Energy spectrum analysis further revealed significant reductions in calcium and phosphorus on the tooth surfaces of *scpp5*^*-/-*^ zebrafish compared to WT (Fig. 2C). Given the fundamental role of calcium and phosphorus in tooth mineralization (Dong et al., 2024), a pharmacological rescue paradigm was employed in *scpp5*^*-/-*^ zebrafish. This involved exposure to high-Ca (30 mM) or high-P (30 mM) egg water to bypass the genetic deficiency via environmental supplementation. Temporal analysis using the *Tg(scpp5:Dendra2-NTR)* line revealed Dendra2-positive cells within the tooth germ at 3 dpf. FISH confirmed that these Dendra2-positive cells co-express endogenous *scpp5* mRNA, validating the specificity of the transgenic reporter for *scpp5*-expressing cells (Fig. S1E, F). According to previous reports, *scpp5*-expressing cells are restricted to IDE cells and mesenchyme-derived odontoblasts (Kawasaki et al., 2021). When reared in high-Ca or high-P egg water at 2-5 dpf, *scpp5*^*-/-*^ zebrafish exhibited a partial rescue of dentin mineralization in response to high-Ca but not high-P, as assessed by alizarin red staining (Fig. 2D and E). Conversely, despite this improvement and the achievement of functional attachment by 5 dpf in high-Ca conditions, the 4V1 enameloid retained alizarin red staining, indicating persistently abnormal mineralization (Fig. 2F). Given that the treatment duration and concentration were selected based on preliminary toxicity tests rather than a fully optimized regimen, these results should be interpreted as a proof-of-principle that calcium supplementation can partially bypass *scpp5* deficiency, while the lack of effect from phosphorus may reflect suboptimal treatment conditions or a genuine biological difference.

To investigate the effects of *scpp5* overexpression on tooth mineralization, the *Tg(hsp70l:scpp5-GFP; cryaa:venus)* zebrafish line was used to achieve temporal overexpression of *scpp5* (Fig. S2A). Temporal overexpression of *scpp5*, either from 2 to 5 dpf or from 3 to 6 dpf, effectively enhanced *scpp5* expression in tooth tissues. However, phenotypic analysis showed that neither short-term (1-day) nor prolonged (3-day) overexpression significantly altered tooth mineralization or tooth germ cell development in WT zebrafish (Fig. S2B-I).

We subsequently used *Tg(hsp70l:scpp5-GFP; cryaa:venus; scpp5*^*-/-*^*)* zebrafish to rescue the *scpp5*^*-/-*^ phenotype (Fig. S3A, B). The results showed that temporal overexpression of *scpp5*, from either 2-5 dpf or 3-6 dpf, promoted dentin mineralization in *scpp5*^*-/-*^ zebrafish under both short-term (1-day) and prolonged (3-day) overexpression conditions (Fig. S3C, D). Specifically, when *scpp5* was overexpressed from 2 to 5 dpf, the 4V1 enameloid was alizarin red-negative in *scpp5*^*-/-*^ zebrafish at 5 dpf. Similarly, when *scpp5* was overexpressed from 3 to 6 dpf, the 4V1 enameloid was alizarin red-negative at 4 dpf (Fig. S3E, F).

### Role of *scpp5* in Tooth Mineralization During Tooth Repair Following Injury

**Fig. 3:**
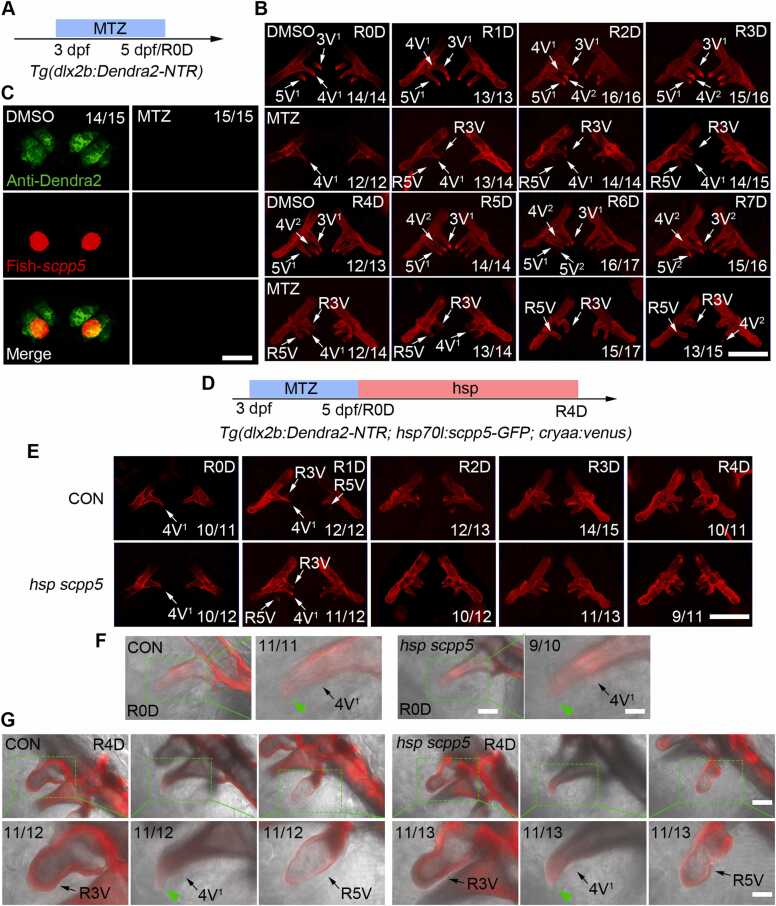
The effect of *scpp5* overexpression to teeth repair mineralization following injury. (A) Experimental schedule. Zebrafish were treated with 12 mM MTZ from 3 to 5 dpf. (B) Three-dimensional reconstruction from Z-stack images of zebrafish teeth under alizarin red staining after treatment with DMSO or MTZ (scale bars, 100 µm). (C) FISH analysis of Dendra2 and *scpp5* after treatment with MTZ (scale bars, 50 µm). (D) Experimental schedule. Overexpression of *scpp5* during R0-R4 D following injury. (E) Three-dimensional reconstruction from Z-stack images of zebrafish teeth under alizarin red staining in the control and hsp *scpp5* group from R0D to R4D (scale bars, 100 µm). (F) Two-dimensional sectioned images of 4V1 under alizarin red staining and brightfield views in the control and hsp *scpp5* group at R0D (scale bars, left 200 µm, right 100 µm). (G) Two-dimensional sectioned images of 4V1, R3V, and R5V under alizarin red staining and brightfield views in the control and hsp *scpp5* group at R4D (scale bars, up 200 µm, down 100 µm). 3 V^1^, the first generation tooth at position 3 in the ventral row; dpf, days post-fertilization; FISH, fluorescence in situ hybridization; MTZ metronidazole; R0D, repair 0 day; R3V, the repair tooth at position 3 in the ventral row; green arrowheads, 4V1 enameloid.

To investigate the function of *scpp5* during tooth repair, the NTR/MTZ system was employed in the *Tg(dlx2b:Dendra2-NTR)* model, previously established in this study, to induce targeted injury of tooth germ cells (Liu et al., 2023). Zebrafish were treated with 12 mM MTZ from 3 to 5 dpf (Fig. 3A). At repair 0 day (R0D), alizarin red staining revealed a complete absence of 3V1 and 5V1 in the MTZ group. During the R0D to R3D period, the repair tooth at position 3 in the ventral row (R3V) and R5V teeth achieved mineralization and functional attachment to the fifth cerato-branchial but displayed significant morphological abnormalities. In contrast, the DMSO control group exhibited a normal progression of 4V2 mineralization. From R3D to R4D, no notable morphological alterations were observed in the MTZ group. Conversely, the DMSO group showed normal 4V2 development, with the tooth progressively advancing toward functional attachment to the fifth cerato-branchial. Between R5D and R7D, the MTZ group experienced gradual detachment of 4V1, while initial mineralization of 4V2 became visible at R7D. No significant changes were detected in R3V and R5V during this period. In the DMSO group, complete attachment of 4V2 to the fifth cerato-branchial was observed, accompanied by the onset of 3V2 and 5V2 mineralization (Fig. 3B). Furthermore, FISH confirmed the ablation of both dental germ cell fluorescence and *scpp5* expression in the MTZ group at R0D (Fig. 3C).

Subsequently, the *Tg(hsp70l:scpp5-GFP; cryaa:venus)* model was used for the overexpression of *scpp5* during the R0D-R4D period to investigate its potential in enhancing tooth repair following injury (Fig. 3D and Fig. S4A). RT-qPCR confirmed successful upregulation of *scpp5* at R1D (Fig. S4B). Although alizarin red staining indicated that *scpp5* overexpression promoted tooth mineralization, the 4V1 enameloid continued to retain alizarin red signal at R0D and R4D, and morphological analysis revealed persistent abnormalities in R3V and R5V (Fig. 3E-G).

### Knockout of *scpp5* Impaired Zebrafish Tooth Mineralization During Development Via Wnt/β-Catenin Signaling

**Fig. 4:**
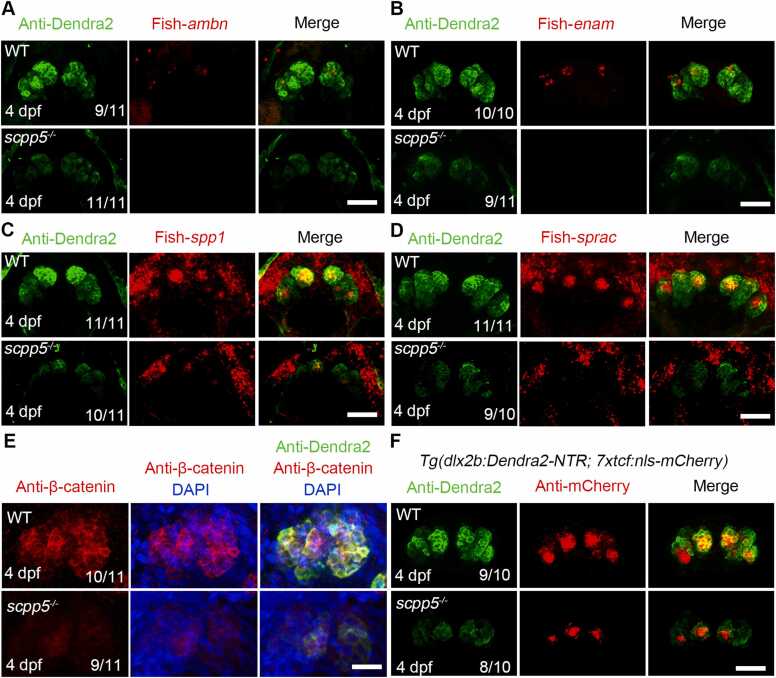
Impact of *scpp5* deficiency on enameloid matrix genes (*ambn*, *eman*), dentin matrix genes (*spp1*, *sprac*), and the Wnt-β-catenin signaling pathway in zebrafish. (A) FISH analysis of Dendra2 and *ambn* at 4 dpf (scale bars, 50 µm). (B) FISH analysis of Dendra2 and *enam* at 4 dpf (scale bars, 50 µm). (C) FISH analysis of Dendra2 and *spp1* at 4 dpf (scale bars, 50 µm). (D) FISH analysis of Dendra2 and *sprac* at 4 dpf (scale bars, 50 µm). (E) Antibody staining of Dendra2 and nucleus β-catenin at 4 dpf (scale bars, 25 µm). (F) Antibody staining of Dendra2 and mCherry at 4 dpf (scale bars, 50 µm). dpf, days post-fertilization.

To elucidate how *scpp5* modulates mineralization in developing zebrafish teeth, the expression of calcium efflux channel genes using RT-qPCR (Qin et al., 2021) was first analyzed. The results revealed significant downregulation of *atp2b1b*, *slc24a3*, *slc24a4a*, and *slc24a4b* in *scpp5*^⁻/⁻^ zebrafish at 4 dpf (Fig. S5A). FISH further revealed reduced expression of the enameloid matrix genes (*ambn* and *enam*) and the dentin matrix genes (*spp1* and *sprac*) in the tooth germ of *scpp5*^⁻/⁻^ zebrafish at 4 dpf (Fig. 4A-D). According to previous studies, *ambn* is expressed in IDE cells; *enam*, in IDE cells and odontoblasts; *spp1*, in odontoblasts and osteoblasts/osteocytes; and *sprac*, in IDE cells, odontoblasts, and osteoblasts/osteocytes (Kawasaki, 2009, Kawasaki et al., 2021).

Given the involvement of multiple signaling pathways in zebrafish tooth mineralization (Gibert et al., 2010, Huysseune et al., 2014, Jackman et al., 2004, Jackman and Gibert, 2020, Jackman et al., 2010, Square et al., 2023, Wise and Stock, 2006, Wise and Stock, 2010), a preliminary screening using RT-qPCR was performed. This screening indicated a significant decrease in the expression of *wnt10a* and *ctnnb1* in *scpp5*^*-/-*^ zebrafish at 4 dpf (Fig. S5B). In zebrafish, β-catenin is encoded by *ctnnb1* and *ctnnb2* (Zhang et al., 2012). Antibody staining revealed reduced nuclear β-catenin protein levels in *scpp5*^⁻/⁻^ zebrafish at 4 dpf (Fig. 4E). Consistent with perturbation of the Wnt/β-catenin pathway, the Wnt/β-catenin reporter line *Tg(7xtcf:nls-mCherry)* (Wang et al., 2024), which drives nls-mCherry expression under 7 TCF-responsive elements, showed decreased mCherry expression in the tooth germ cells of *scpp5*^⁻/⁻^ zebrafish at 4 dpf (Fig. 4F). Additionally, RT-qPCR demonstrated a marked decline in the expression of Wnt/β-catenin target genes (*axin2*, *c-myc*, and *lef1*) in the tooth germ cells of *scpp5*^⁻/⁻^ zebrafish at 4 dpf (Fig. S5C).

**Fig. 5:**
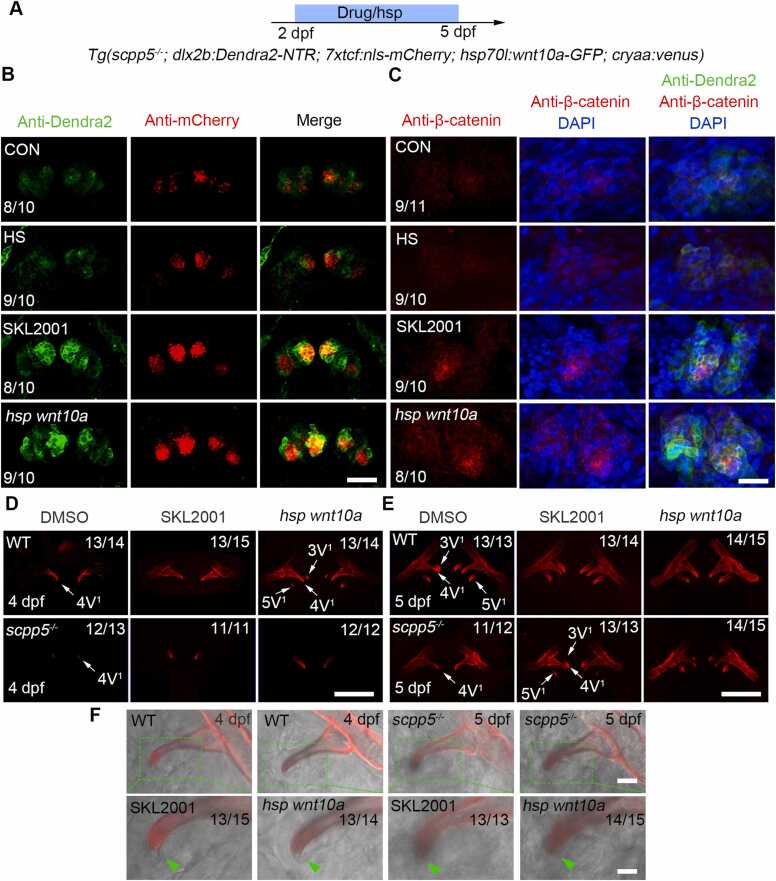
Effects of activating the Wnt-β-catenin signaling pathway on tooth mineralization in WT and *scpp5*^*-/-*^ zebrafish. (A) Experimental schedule. (B) Antibody staining of Dendra2 and mCherry at 4 dpf after activating Wnt-β-catenin signaling (scale bars, 50 µm). (C) Antibody staining of Dendra2 and nucleus β-catenin at 4 dpf after activating Wnt-β-catenin signaling (scale bars, 25 µm). (D, E) Three-dimensional reconstruction from Z-stack images of zebrafish teeth under alizarin red staining after activating Wnt-β-catenin in WT and *scpp5*^*-/-*^zebrafish at 4 and 5 dpf (scale bars, 100 µm). (F) Two-dimensional sectioned images of 4V1 under alizarin red staining and brightfield views after activating Wnt-β-catenin in *scpp5*^*-/-*^zebrafish at 4 and 5 dpf (scale bars, up 200 µm, down 100 µm). 3 V^1^, the first generation-tooth at position 3 in the ventral row; dpf, days post-fertilization; green arrowheads, 4V1 enameloid; HS, heat-shock.

**Fig. 6:**
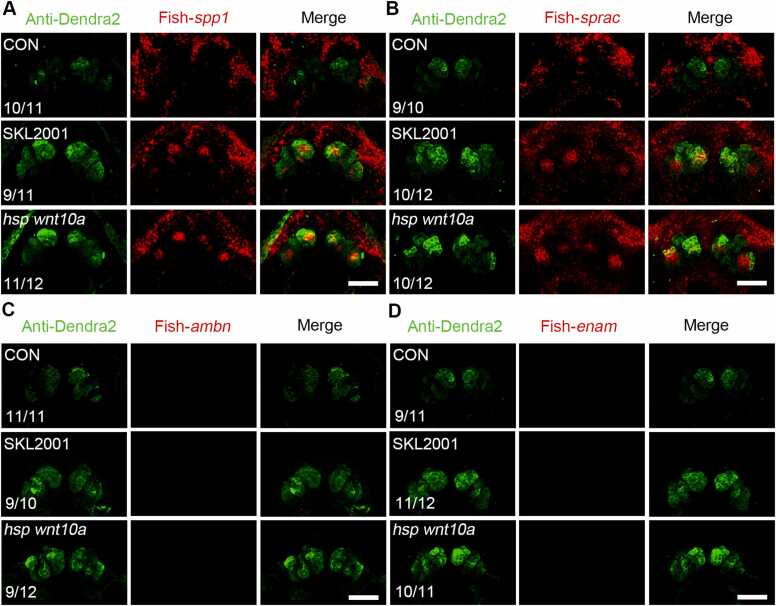
Effects of activating the Wnt-β-catenin signaling pathway on enameloid matrix genes (*ambn*, *eman*) and dentin matrix genes (*spp1*, *sprac*) in zebrafish. (A) FISH analysis of Dendra2 and *spp1* after activating Wnt/β-catenin signaling in *scpp5*^*-/-*^ zebrafish at 4 dpf (scale bars, 50 µm). (B) FISH analysis of Dendra2 and *sprac* after activating Wnt/β-catenin signaling in *scpp5*^*-/-*^ zebrafish at 4 dpf (scale bars, 50 µm). (C) FISH analysis of Dendra2 and *ambn* after activating Wnt/β-catenin signaling in *scpp5*^*-/-*^ zebrafish at 4 dpf (scale bars, 50 µm). (D) FISH analysis of Dendra2 and *enam* after activating Wnt/β-catenin signaling in *scpp5*^*-/-*^ zebrafish at 4 dpf (scale bars, 50 µm).

Subsequently, the Wnt/β-catenin signaling was activated pharmacologically using SKL2001 and genetically using the *Tg(hsp70l:wnt10a-GFP; cryaa:venus)* line (Figs. 5A and S5D) (Gwak et al., 2011). The *hsp:wnta10a* group successfully upregulated *wnt10a* expression, but not the SKL2001 group or the heat-shock control group (Fig. S5E). Notably, both tooth germ cell development and Wnt/β-catenin signaling were enhanced in *scpp5*^⁻/⁻^ zebrafish in the *hsp:wnt10a* and SKL2001 groups, whereas no changes were observed in the heat-shock group (Figs. 5B, C and S5F). Furthermore, both SKL2001 and *hsp:wnt10a* enhanced dentin mineralization in WT and *scpp5*^*⁻/⁻*^ zebrafish (Fig. 5D, E). Moreover, both treatments upregulated *spp1* and *sprac* expression at 4 dpf in *scpp5*^*⁻/⁻*^ zebrafish. However, the 4V1 enameloid continued to exhibit alizarin red signal in both treatment groups at 5 dpf, and the expression of *ambn* and *enam* remained unchanged from 3 to 5 dpf (Figs. 5F, 6A-D, and S6A-D). In *scpp5*^*⁻/⁻*^ zebrafish, both SKL2001 and *hsp:wnt10a* partially rescued the downregulation of specific calcium efflux channel genes (*slc24a3*, *slc24a4a*, and *slc24a4b*), whereas neither treatment significantly affected *atp2b1b* expression (Fig. S6E).

### *scpp5* Overexpression Accelerated Tooth Mineralization During Repair by Activating the Wnt/β-Catenin Pathway

**Fig. 7:**
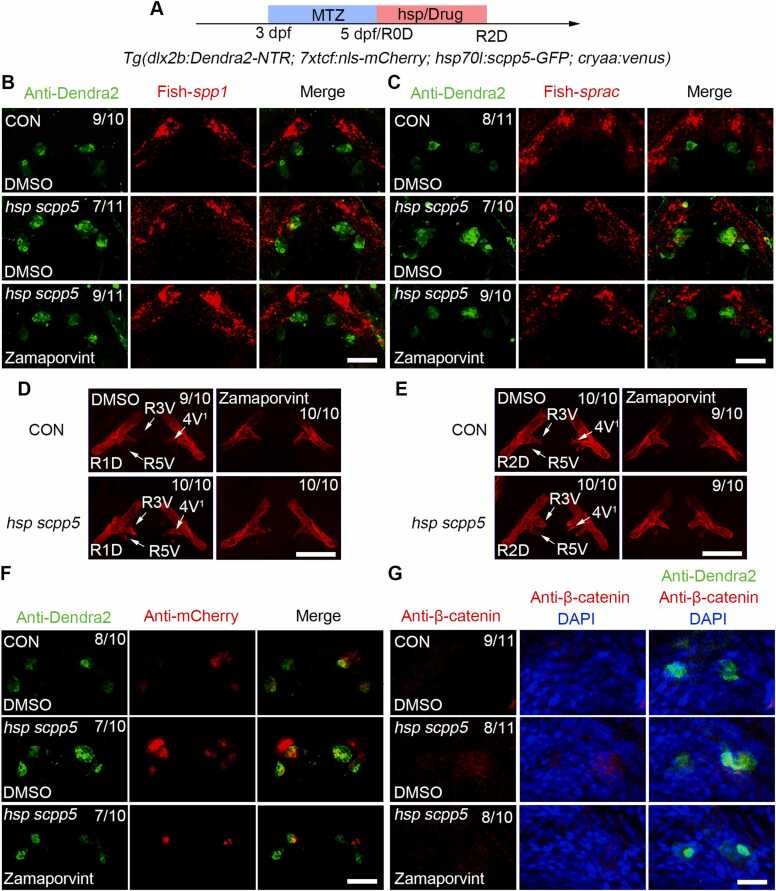
Overexpression of *scpp5* promotes zebrafish tooth mineralization repair following injury via Wnt/β-catenin signaling. (A) Experimental schedule. Inhibition of Wnt-β-catenin signaling by drug (Zamaporvint) treatment during R0D-R2D following injury. (B) FISH analysis of Dendra2 and *spp1* at R1D (scale bars, 50 µm). (C) FISH analysis of Dendra2 and *sprac* at R1D (scale bars, 50 µm). (D, E) Three-dimensional reconstruction from Z-stack images of zebrafish teeth under alizarin red staining after Inhibiting Wnt-β-catenin in the control and *hsp scpp5* group at R1D and R2D (scale bars, 100 µm). (F) Antibody staining of Dendra2 and mCherry at R1D (scale bars, 50 µm). (G) Antibody staining of Dendra2 and nucleus β-catenin at R1D (scale bars, 25 µm). 4 V^1^, the first generation-tooth at position 4 in the ventral row; dpf, days post-fertilization; MTZ metronidazole; R0D, repair 0 day; R3V, the repair tooth at position 3 in the ventral row.

During tooth repair following injury, overexpression of *scpp5* significantly upregulated the expression of specific calcium efflux channel genes (*atp2b4, slc8a1a*, *slc8a3*, *slc24a3*, and *slc24a4b*) and dentin matrix genes (*spp1* and *sprac*) at R1D (Figs. 7A-C and S7A). Furthermore, it enhanced the repair of tooth germ cells and tooth mineralization at R1D (Fig. 7B-E). In contrast, the 4V1 and 4V2 tooth germs failed to develop in either group, with no concomitant changes in *ambn* or *enam* expression at R1D (Fig. S7B and C). To investigate the potential involvement of Wnt/β-catenin signaling in this process, the pathway was inhibited pharmacologically using Zamaporvint (Fig. 7A) (Phillips et al., 2022). The results showed that overexpression of *scpp5* could enhance the repair of tooth germ cells and the signaling of Wnt/β-catenin at R1D, effects that were attenuated by Zamaporvint treatment (Figs. 7F, G, and S6D, E). Furthermore, Zamaporvint inhibited tooth mineralization in both the control and *hsp:scpp5* group zebrafish at R1D and R2D (Fig. 7D and E). Consistent with this phenotype, the expression of the specific calcium efflux channel genes (*atp2b4*, *slc8a1a*, *slc8a3*, *slc24a3*, and *slc24a4b*) decreases following Zamaporvint treatment at R1D (Fig. S7F).

## DISCUSSION

There exists a profound and complex relationship between organismal development and repair following injury, which share fundamental mechanisms, even though repair often fails to faithfully recapitulate the developmental process (Gurtner et al., 2008). The mineralization of teeth during injury repair is a precisely coordinated process that recapitulates functional layered structures, demonstrating conserved molecular pathways with developmental mineralization (Sui et al., 2023, Zhang and Yelick, 2021). A dual role of scpp5 in zebrafish tooth mineralization was defined in the present study. During development, *scpp5* knockout suppressed dentin mineralization by inhibiting Wnt/β-catenin signaling. Conversely, while *scpp5* overexpression did not affect dentin developmental mineralization, it markedly enhanced dentin mineralization during injury-induced repair by activating the Wnt/β-catenin pathway.

Compared to WT zebrafish, *scpp5*^*-/-*^ zebrafish exhibited abnormal enameloid mineralization during tooth development, confirming that *scpp5* belongs to the P/Q-rich SCPP family involved in enamel or enameloid formation (Kawasaki and Weiss, 2008). The differential spatio-temporal expression profiles of *dlx2b* (detectable at 2 dpf) and *scpp5* (emerging at 3 dpf) in zebrafish dental mesenchyme and epithelium reveal a developmental hierarchy, positioning dlx2b as a pioneer marker for primitive odontogenic progenitors prior to mineralization-related *scpp5* activation (Jackman and Stock, 2006). The observation that WT zebrafish tooth mineralization commenced at 3 dpf temporally confined the window of SCPP5-mediated regulation to post-2 dpf stages, during which *scpp5* knockout exerted its principal effects on tooth germ cells development and subsequent mineralization processes. In this study, *scpp5* knockout attenuated *dlx2b* expression during zebrafish tooth development. In contrast, *scpp5* overexpression exhibited context-dependent regulation: it was neutral during development but specifically potentiated the induction of *dlx2b* during injury-triggered mineralization repair. Emerging genetic evidence reveals that while *dlx3b* knockout downregulates *scpp5* expression in zebrafish tooth (Pang et al., 2020), the potential regulatory influence of *dlx2b* on *scpp5* remains unexplored. This incomplete reciprocity suggests an unexpectedly complex, multi-nodal regulatory circuitry between *scpp* and *dlx* gene families that challenges conventional linear pathway models and warrants systematic interrogation.

Biomineralization in zebrafish and mammalian teeth similarly involve calcium secretion from tooth germ cells, which actively contributes to extracellular matrix mineralization (Liu and Yang, 2025). Proper regulation of calcium efflux channel genes is essential for controlled biomineralization. The findings of the present study demonstrate that *scpp5* modulated distinct calcium efflux channel genes during developmental versus repair mineralization processes in the zebrafish tooth. Quantitative elemental analysis revealed impaired calcium accumulation on *scpp5*^*-/-*^ zebrafish tooth surfaces, paralleled by transcriptional suppression of specific calcium efflux channel genes (*atp2b1b*, *slc24a3*, *slc24a4a*, and *slc24a4b*). As members of the *slc24a* gene family, *slc24a3*, *slc24a4a*, and *slc24a4b* encode K⁺-dependent Na⁺/Ca²⁺ exchangers mediate Ca²⁺ efflux to participate in tooth mineralization (Al-Khannaq and Lytton, 2022). Comparative genomic analysis reveals that the zebrafish *slc24a4a* and *slc24a4b* paralogs correspond to a single ortholog (*Slc24a4*) in mammals. Genetic ablation of *Slc24a4* in mice results in profound enamel mineralization defects, accompanied by a 66% decrease in intracellular Ca²⁺ concentration within ameloblasts (Parry et al., 2013). Furthermore, clinical characterization in humans with homozygous *SLC24A4* mutations results in yellowish-brown teeth that exhibit irregular pits, severe attrition, and malocclusion. Radiographic examination confirms evidence of mineralization defects (Lepperdinger et al., 2020). Although *Atp2b1b* encodes plasma membrane Ca^2+^ -ATPase (PMCA) belonging to the *Atp2b* transporter family (Nurbaeva et al., 2016), no experimental evidence has yet established its involvement in tooth mineralization processes. During injury-induced repair, *scpp5* overexpression upregulated the expression of specific calcium efflux channel genes including *atp2b4*, *slc8a1a*, *slc8a3*, *slc24a3*, and *slc24a4b*. *atp2b4* belongs to the *Atp2b* family, while *slc8a1a* and *slc8a3* are members of the *Slc8a* family that mediate direct Na⁺/Ca²⁺ exchange to extrude Ca^2+^ during tooth mineralization (Iwamoto and Kita, 2006). Tooth biomineralization is governed by an intricate Ca²⁺ regulatory network. Current evidence suggests that *scpp5* participates in coordinating Ca²⁺ homeostasis, yet its exact regulatory mechanisms—particularly the spatiotemporal control of calcium transporters during developmental versus regenerative mineralization—require further elucidation.

A particularly intriguing finding is that, although pharmacological or genetic activation of Wnt/β-catenin signaling and calcium supplementation rescued the dentin mineralization defect (assessed by alizarin red staining for calcium deposits) in *scpp5*⁻/⁻ zebrafish, none of these interventions normalized the abnormal alizarin red staining pattern within the enameloid matrix. This dissociation points to 2 separable functions of *scpp5*, First, *scpp5* regulates calcium efflux through Wnt/β-catenin signaling to promote hydroxyapatite crystal growth during dentin mineralization. Second, as a member of the SCPP family**—**which is characterized by multiple phosphorylated serine residues that can directly bind calcium ions (Kawasaki and Weiss, 2003)**—***scpp5* may directly participate in enameloid mineralization by coordinating Ca²⁺ to facilitate hydroxyapatite nucleation. This functional duality likely reflects distinct cellular origins: dental epithelial cells, which secrete enameloid matrix proteins, may require *scpp5* for matrix organization (as reflected by alizarin red staining), whereas odontoblast-lineage cells may utilize *scpp5* to activate Wnt signaling for calcium transport. Future studies employing cell-type-specific rescue approaches are needed to dissect these lineage-dependent roles.

A methodological note regarding the use of the *hsp70l* global overexpression driver in the tooth injury model is warranted. The MTZ/NTR system ablates the entire tooth germ, including both epithelial and mesenchymal compartments, as evidenced by the complete loss of Dendra2 and *scpp5* expression at R0D (Fig. 3C). Consequently, the cell type (epithelium vs. mesenchyme) or tooth position (4V1 vs 3V1/5V1) that limits repair cannot be predicted a priori. The repairing tooth germ represents a heterogeneous, multicellular structure, and the use of a global heat-shock promoter enabled an unbiased assessment of whether *scpp5* overexpression in any tooth germ cell type could promote repair. Our observation that *scpp5* overexpression specifically accelerated mineralization in 3V1 and 5V1 (mesenchymal-derived dentin) but not in 4V1 (epithelial-derived enameloid) revealed a cell type- and tooth position-specific effect despite global overexpression (Fig. 3E-G). Future studies employing cell-type-specific drivers will further dissect the cell-autonomous versus non-cell-autonomous roles of scpp5 during tooth repair.

A conceptual question arises: how does SCPP5, a secreted extracellular matrix protein, regulate the expression of tooth-related genes? We propose an **"**outside-in" signaling feedback loop rather than direct DNA binding or transcriptional activation. As a major component of the enameloid extracellular matrix, SCPP5 helps organize the matrix framework for hydroxyapatite crystal deposition (Kawasaki et al., 2021). The composition and integrity of this extracellular matrix are continuously monitored by cells via integrins, focal adhesions, and other cell-surface receptors. When SCPP5 is absent, the extracellular matrix is structurally compromised—as suggested by the persistent alizarin red signal in *scpp5*^*-/-*^ zebrafish at 5 dpf (Fig. 1E)—which may disrupt normal cell-matrix adhesion and tension. This mechanical perturbation can be transduced into the nucleus via mechanotransduction pathways (eg, integrin-FAK-MAPK), ultimately altering transcription factor activity and gene expression. This "outside-in" signaling mechanism is well documented for other secreted matrix proteins in tooth development. For example, the secreted matrix protein DPP binds to integrin αVβ1, activating FAK phosphorylation and the MAPK/ERK cascade to regulate odontoblast differentiation and *Dspp* expression (Eapen et al., 2012). Similarly, *Ambn* knockout in mice leads to downregulation of enamel matrix genes (*Amelx*, *Enam*) in ameloblasts through disrupted cell-matrix feedback (Fukumoto et al., 2004). DMP1 also functions as an extracellular signaling molecule that regulates odontoblast differentiation via integrin binding (Almushayt et al., 2006, von Marschall and Fisher, 2008, Wu et al., 2011). Thus, we propose that SCPP5, by organizing the enameloid extracellular matrix, provides critical feedback signals that maintain the differentiation state of enameloid-forming cells and coordinate the expression of calcium efflux channels and matrix genes during both development and repair.

The development of 4 teeth—3V1, 4V1, 5V1, and 4V2—was affected by MTZ treatment (administered at 3-5 dpf), with outcomes varying according to each tooth's developmental stage at the time of treatment: As the first tooth to erupt in zebrafish, 4V1 begins development at 48 hpf. After MTZ exposure, 4V1 exhibited normal morphology, with only enameloid mineralization at the tip being impaired. The development of 3V1 and 5V1 begins after that of 4V1 (56 hpf) (Laurenti et al., 2004). At R0D, these 2 teeth showed no obvious mineralization, and subsequent mineralization repair led to teeth with abnormal morphology and a lack of a well-defined tip. 4V2, which starts developing around 80 hpf (Laurenti et al., 2004), displayed no detectable mineralization from R0D to R6D following MTZ treatment. Although a newly mineralized tooth was observed at R7D, it remains challenging to definitively determine whether this represents delayed mineralization of the pre-existing 4V2 tooth germ (which normally begins mineralizing at R2D in the DMSO group) or premature emergence of the next-generation 4V3 tooth, given the continuous polyphyodont replacement in zebrafish. This ambiguity represents a limitation of the current study, as our experimental design lacks single-tooth lineage tracing. Future studies employing in *vivo* time-lapse imaging or genetic tooth-specific labeling are required to resolve this uncertainty.

The Wnt signaling pathway is a crucial regulator of tooth formation in oral tissue development and disease (Liu and Millar, 2010). However, its role in regulating zebrafish tooth organogenesis remains debated (Alhazmi et al., 2021, Benard and Hammerschmidt, 2025, Huysseune et al., 2014, Shim et al., 2018, Square et al., 2023). Among Wnt ligands, *wnt10a* is expressed in both the dental mesenchyme and epithelium during zebrafish tooth development (Benard and Hammerschmidt, 2025, Benard et al., 2023). Both morpholino-mediated knockdown and genetic mutation of wnt10a result in impaired tooth development at 5 dpf (Yuan et al., 2017), and adult *wnt10a* mutants lack teeth in the fifth cerato-branchial (Benard et al., 2023). Conversely, several studies have shown that constitutive activation of Wnt signaling can also lead to tooth absence, delayed formation, or hypo-mineralization in zebrafish (Huysseune et al., 2014, Song and Kim, 2025). In the present study, activating Wnt/β-catenin signaling promoted tooth development, whereas its inhibition impaired injury-induced tooth repair. These findings highlight the context-dependent complexity of canonical Wnt signaling, where outcomes may vary depending on the specific gene involved, signaling dosage, developmental timing, or physiological context (Huysseune et al., 2014, Shim et al., 2018).

Zebrafish have emerged as a powerful model for dissecting the genetic control of tooth renewal. Although human *scpp5* is a pseudogene, the functional module identified in this study—comprising a secreted phosphoprotein (*scpp5*) and the canonical Wnt pathway—is directly relevant to human dental disorders. In humans, mutations in other acidic SCPPs (eg, *AMELX*, *ENAM*, *ODAM*) cause enamel and dentin defects that phenocopy the *scpp5* mutant zebrafish phenotype. Furthermore, this study’s observation that Wnt activation enhances post-injury repair aligns with mammalian studies demonstrates that Wnt signaling promotes dental mesenchymal stem cell differentiation. Thus, the zebrafish *scpp5* loss-of-function model serves as a proxy for understanding how dysregulation of the SCPP-Wnt axis leads to mineralization defects and suggests that targeting Wnt/β-catenin signaling could be a viable strategy for stimulating human dentin/enamel repair.

As one of the extracellular matrix proteins deposited by tooth germ cells, *scpp5* is indispensable for normal tooth development, as its knockout impairs mineralization (Kawasaki et al., 2021). However, *scpp5* overexpression does not affect physiological mineralization but does promotes injury-induced repair. We propose a "saturation versus limitation" model to explain this context-dependent role. During normal development, endogenous *scpp5* expression in the enameloid epithelium is already saturated; therefore, additional *scpp5* provided by the *hsp70l* overexpression construct cannot further accelerate or enhance mineralization (Fig. S2). However, *scpp5* remains necessary because its knockout cannot be compensated by other SCPP family members, leading to impaired calcium efflux and downregulation of enameloid and dentin matrix genes (Fig. 4). During injury repair, MTZ/NTR ablation destroys the entire tooth germ, creating a functional deficit of *scpp5* (Fig. 3C). In this context, *scpp5* becomes a limiting factor for mineralization repair, particularly in mesenchymal-derived dental tissues (3V1 and 5V1). Exogenous *scpp5* overexpression can now rescue this deficit and promote repair (Fig. 3E-G). Mechanistically, the differential engagement of Wnt/β-catenin signaling further supports this context-dependent role. During development, Wnt activation rescued calcium channel expression and dentin matrix genes but failed to restore *ambn/enam* or enameloid mineralization (Figs. 5D-F and 6C, D), suggesting that *scpp5* may regulate enameloid matrix genes through a Wnt-independent pathway. During repair, *scpp5* overexpression actively upregulated Wnt/β-catenin signaling (Fig. 7F-G), and pharmacological inhibition of Wnt blocked *scpp5*-mediated repair (Fig. 7D, E), indicating that in the repair context, *scpp5* functions upstream of Wnt/β-catenin to promote dentin mineralization.

## CONCLUSION

In summary, *scpp5* loss suppresses tooth mineralization during zebrafish development by inhibiting Wnt/β-catenin signaling, whereas *scpp5* overexpression promotes mineralization during injury-induced repair by activating the same pathway. These findings reveal a context-dependent dual role for *scpp5* and highlight the SCPP5-Wnt/β-catenin axis as a potential therapeutic target for dental tissue engineering.

## CRediT authorship contribution statement

**Qiqi Liu:** Writing – original draft, Validation, Methodology, Investigation. **Weifeng Hao:** Writing – original draft, Software, Methodology, Data curation, Conceptualization. **Zhenan Zhang:** Supervision, Software, Formal analysis, Data curation, Conceptualization. **Yu Yue:** Visualization, Validation, Investigation, Formal analysis. **Deqin Yang:** Writing – review & editing, Methodology, Funding acquisition, Data curation.

## Declaration of Competing Interests

The authors declare that they have no conflict of interest.

## Appendix A Supplementary material

**Fig. S1** The effect of knockout *scpp5* on the zenrafish and the *Tg(scpp5:Dendra2-NTR).* (A) The gel electrophoresis showed amplification of the SCPP5 coding sequence using the same primer pair for both WT and *scpp5*^*-/-*^ zebrafish cDNA at 3 dpf. (B) The heat-shock of *Tg(hsp70l: scpp5*^*+/+*^*-p2a-DsRed; cryaa:venus)* and *Tg(hsp70l: scpp5*^*-/-*^*-p2a-DsRed; cryaa:venus)* (scale bars, 1 mm). (C) The representative tooth mineralization absence in *scpp5*^*-/-*^ zebrafish at 9 dpf. (a) unilateral 3V1 mineralization absence; (b) unilateral 3V1 and 4V1 mineralization absence; (c) bilateral 4V1 mineralization absence (scale bars, 200 µm). (D) The 3D rendered micro-CT images of the skeleton in WT and *scpp5*^*-/-*^ adult zebrafish (scale bars, 5 mm). (E) Schematic illustration of *Tg(scpp5:Dendra2-NTR)*. (F) FISH analysis of Dendra2 and *scpp5* expression in *Tg(scpp5:Dendra2-NTR)* zebrafish from 2 to 4 dpf. 3 V^1^, the first generation-tooth at position 3 in the ventral row; dpf, days post-fertilization; mpf, months post-fertilization; WT, wild type.

**Fig. S2** Effects of *scpp5* overexpression on mineralization and tooth germ development in zebrafish. (A) Schematic illustration of *Tg(hsp70l:scpp5-GFP; cryaa:venus)*. (B, C) Experimental schedule. Overexpression of *scpp5* during 2 to 5 dpf and 3 to 6 dpf. (D, E) RT-qPCR showed *scpp5* expression after overexpression of *scpp5* during 2 to 5 dpf and 3 to 6 dpf. (F, G) Three-dimensional reconstruction from Z-stack images of zebrafish teeth under alizarin red staining after overexpression of *scpp5* during 2 to 5 dpf and 3 to 6 dpf (scale bars, 100 µm). (H, I) Antibody staining of Dendra2 after overexpression of *scpp5* during 2 to 5 dpf and 3 to 6 dpf (scale bars, 50 µm). The bar graph presents the mean and standard deviation, *P* value *** *P* < .01 and **** *P* < .0001 were calculated by one-way ANOVA. dpf, days post-fertilization.

**Fig. S3** Restoration of tooth mineralization by scpp5 overexpression in *scpp5*^*-/-*^ zebrafish. (A, B) Experimental schedule. Overexpression of *scpp5* during 2 to 5 dpf and 3 to 6 dpf in *scpp5*^*-/-*^ zebrafish. (C, D) Three-dimensional reconstruction from Z-stack images of zebrafish teeth under alizarin red staining after overexpression of *scpp5* during 2 to 5 dpf and 3 to 6 dpf (scale bars, 100 µm). (E) Two-dimensional sectioned images of 4V1 under alizarin red staining and brightfield views in *hsp scpp5* group from at 5 dpf (scale bars, left 200 µm, right 100 µm). (F) Two-dimensional sectioned images of 4V1 under alizarin red staining and brightfield views in *hsp scpp5* group from at 4 dpf (scale bars, left 200 µm, right 100 µm). dpf, days post-fertilization.

**Fig. S4** The effect of *Tg(hsp70l:scpp5-GFP; cryaa:venus)* on the expression of *scpp5.* (A) In vivo fluorescence images of *Tg(dlx2b:Dendra2-NTR; hsp70l:scpp5-GFP; cryaa:venus)* zebrafish with or without MTZ and heat-shock treatment at R0D. (B) RT-qPCR showed *scpp5* expression after overexpression of *scpp5* at R1D (scale bars, 1 mm). The bar graph presents the mean and standard deviation, *P* value ** *P* < .01 was calculated by *t*-test. MTZ metronidazole; R1D, repair 1 day.

**Fig. S5** Effects of *scpp5* knockout on gene expression and validation of Wnt/β-catenin pathway activation in zebrafish. (A) RT-qPCR showed the expression of genes related to calcium efflux channel in WT and *scpp5*^*-/-*^ zebrafish at 4 dpf. (B) RT-qPCR showed the expression of signaling pathway genes in WT and *scpp5*^*-/-*^ zebrafish at 4 dpf. (C) RT-qPCR showed the expression of Wnt/β-catenin signaling target genes (*axin2*, *c-myc*, and *lef1*) in WT and *scpp5*^*-/-*^ zebrafish at 4 dpf. (D) In vivo fluorescence images of *Tg(hsp70l:wnt10a-GFP; cryaa:venus)* with or without heat-shock at 2dpf (scale bars, 1 mm). (E) RT-qPCR showed the expression of *wnt10a* at 4 dpf after activating Wnt-β-catenin signaling. (F) RT-qPCR showed the expression of Wnt/β-catenin signaling target genes (*axin2*, *c-myc*, and *lef1*) after activating Wnt/β-catenin signaling in *scpp5*^*-/-*^ zebrafish at 4 dpf. The bar graph presents the mean and standard deviation, *P* value ns, not significant, * *P* < .05, ** *P* < .01, *** *P* < .001, and **** *P* < .0001 were calculated were calculated by *t*-test (A-C) or one-way ANOVA (E, F). dpf, days post-fertilization.

**Fig. S6** Effects of *scpp5* knockout and Wnt/β-catenin pathway activation on enameloid matrix gene expression and calcium efflux channel genes in zebrafish. (A) FISH analysis of *ambn* expression in WT, *scpp5*^*-/-*^, and *scpp5*^*-/-*^ zebrafish following activation of the Wnt/β-catenin signaling pathway at 3 dpf (scale bars, 50 µm). (B) FISH analysis of enam expression in WT, *scpp5*^*-/-*^, and *scpp5*^*-/-*^ zebrafish following activation of the Wnt/β-catenin signaling pathway at 3 dpf (scale bars, 50 µm). (C) FISH analysis of *ambn* expression in WT, *scpp5*^*-/-*^, and *scpp5*^*-/-*^ zebrafish following activation of the Wnt/β-catenin signaling pathway at 5 dpf (scale bars, 50 µm). (D) FISH analysis of enam expression in WT, *scpp5*^*-/-*^, and *scpp5*^*-/-*^ zebrafish following activation of the Wnt/β-catenin signaling pathway at 5 dpf (scale bars, 50 µm). (E) RT-qPCR analysis showing that activation of the Wnt/β-catenin pathway modulates the expression of specific calcium efflux channel genes that are downregulated in *scpp5*^*-/-*^ zebrafish compared to WT at 4 dpf. The bar graph presents the mean and standard deviation, *p* value ns, not significant, * *P* < .05, ** *P* < .01, *** *P* < .001, and **** *P* < .0001 were calculated were calculated by one-way ANOVA. dpf, days post-fertilization; hsp, *hsp wnt10a*; SKL, SKL2001.

**Fig. S7** Effects of *scpp5* overexpression on calcium efflux channel genes, enameloid matrix genes, and the Wnt/β-catenin signaling pathway at R1D, and modulation by pathway inhibition. (A) RT-qPCR analysis of calcium efflux channel-related gene expression at R1D following *scpp5* overexpression. (B) FISH analysis of Dendra2 and *ambn* expression at R1D following *scpp5* overexpression (scale bars, 50 µm). (C) FISH analysis of Dendra2 and *enam* expression at R1D following *scpp5* overexpression (scale bars, 50 µm). (D) RT-qPCR analysis of *wnt10a* expression at R1D. (E) RT-qPCR analysis of Wnt/β-catenin target gene expression (*axin2*, *c-myc*, *lef1*) at R1D following *scpp5* overexpression. (F) RT-qPCR analysis showing that Wnt/β-catenin pathway inhibition modulates the expression of specific calcium efflux channel genes that are upregulated upon *scpp5* overexpression at R1D.

## Footnotes

Appendix A Supplementary data associated with this article can be found in the online version at doi:10.1016/j.mocell.2026.100377.
