# Supplementary figures and images for: scpp5 regulates tooth development and injury-induced repair in zebrafish through mineralization and Wnt/β-catenin signaling
Source: Mol Cells. 2026 Jun 3;49(8):100377. doi: 10.1016/j.mocell.2026.100377 (PMC13312475; doi:10.1016/j.mocell.2026.100377)

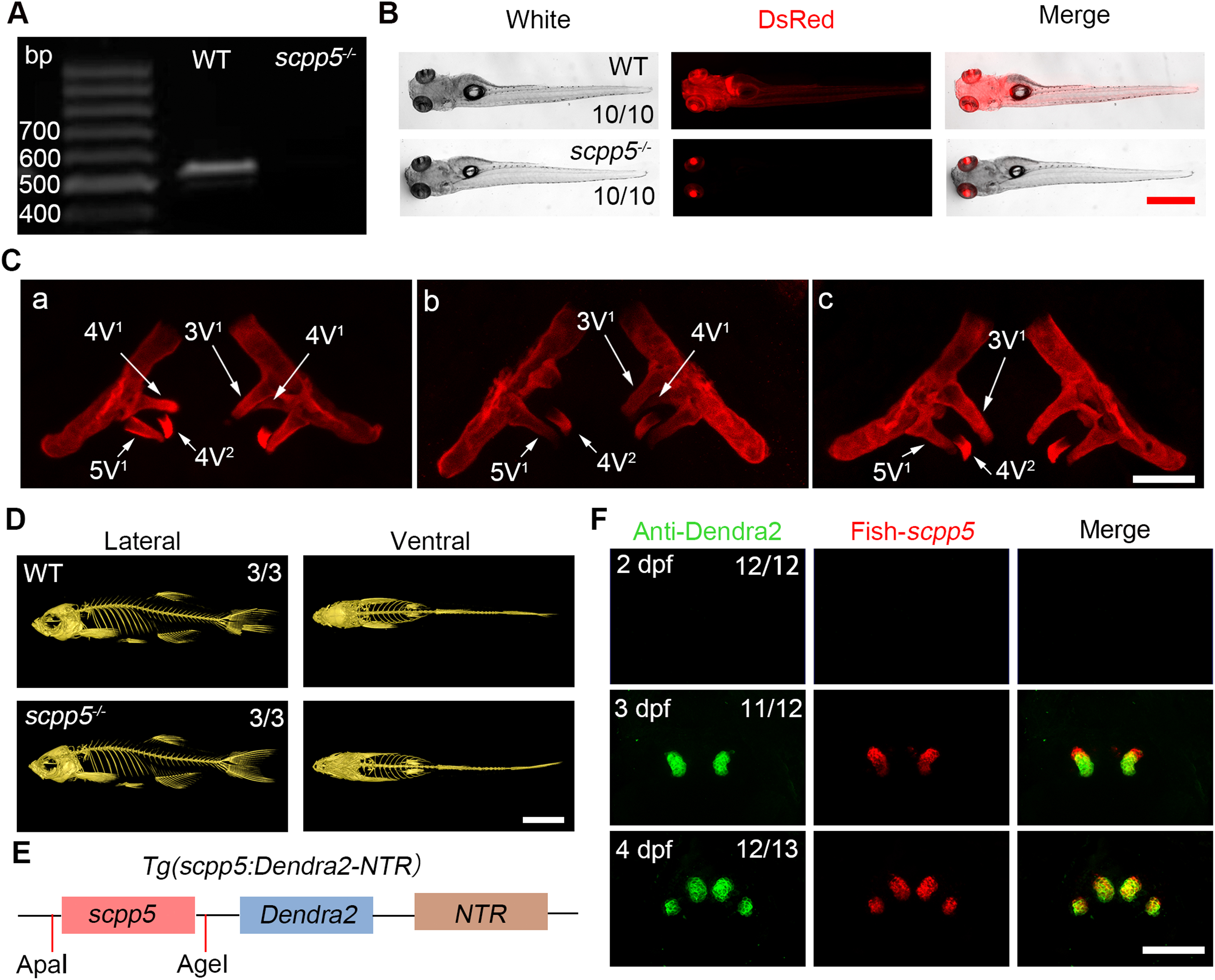

Supplement: Supplementary file 1 — Fig. S1 The effect of knockout scpp5 on the zenrafish and the Tg(scpp5:Dendra2-NTR). (A) The gel electrophoresis showed amplification of the SCPP5 coding sequence using the same primer pair for both WT and scpp5-/- zebrafish cDNA at 3 dpf. (B) The heat-shock of Tg(hsp70l: scpp5+/+-p2a-DsRed; cryaa:venus) and Tg(hsp70l: scpp5-/--p2a-DsRed; cryaa:venus) (scale bars, 1 mm). (C) The representative tooth mineralization absence in scpp5-/- zebrafish at 9 dpf. (a) unilateral 3V1 mineralization absence; (b) unilateral 3V1 and 4V1 mineralization absence; (c) bilateral 4V1 mineralization absence (scale bars, 200 µm). (D) The 3D rendered micro-CT images of the skeleton in WT and scpp5-/- adult zebrafish (scale bars, 5 mm). (E) Schematic illustration of Tg(scpp5:Dendra2-NTR). (F) FISH analysis of Dendra2 and scpp5 expression in Tg(scpp5:Dendra2-NTR) zebrafish from 2 to 4 dpf. 3 V1, the first generation-tooth at position 3 in the ventral row; dpf, days post-fertilization; mpf, months post-fertilization; WT, wild type. [file mmc1.jpg]

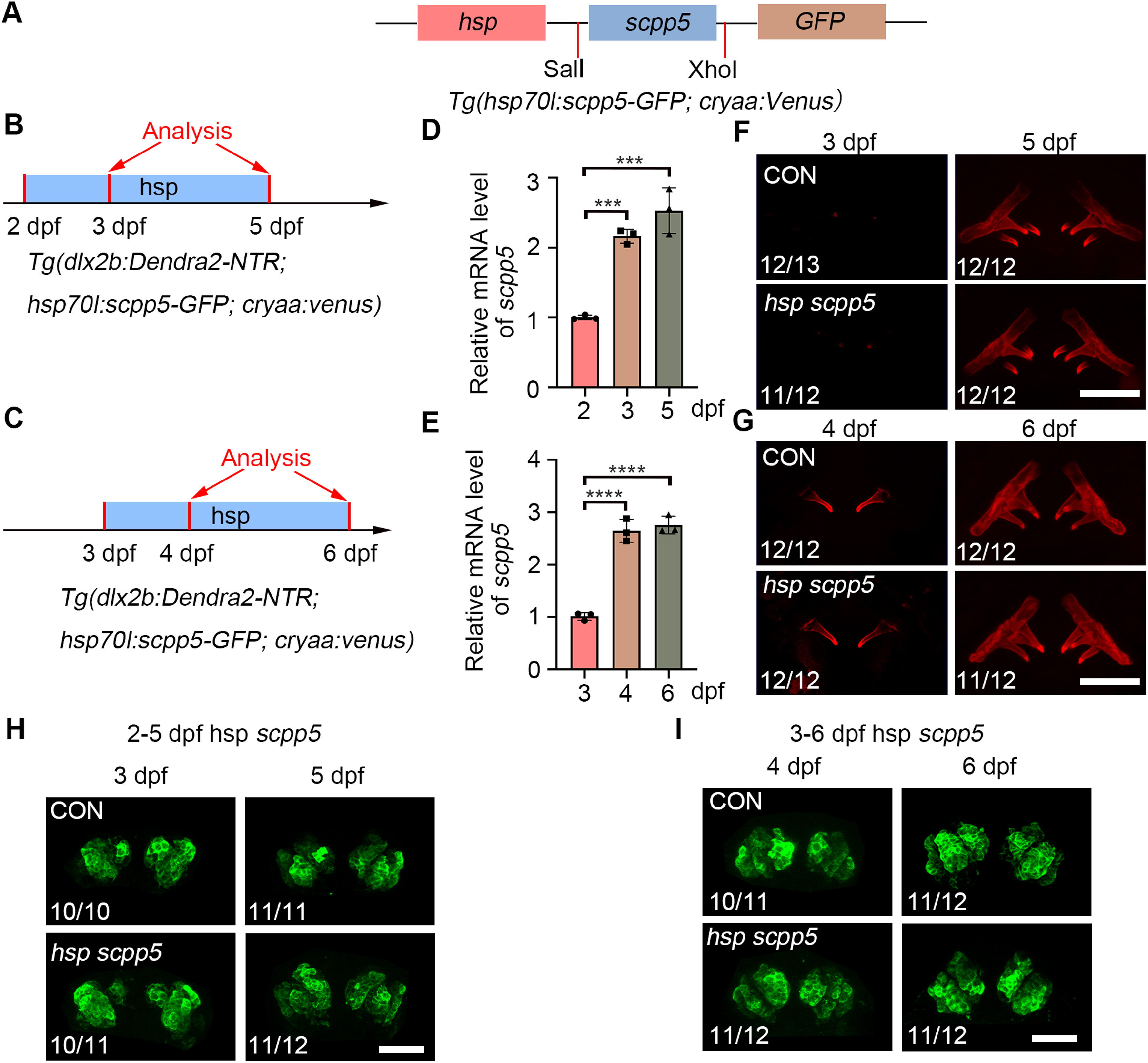

Supplement: Supplementary file 2 — Fig. S2 Effects of scpp5 overexpression on mineralization and tooth germ development in zebrafish. (A) Schematic illustration of Tg(hsp70l:scpp5-GFP; cryaa:venus). (B, C) Experimental schedule. Overexpression of scpp5 during 2 to 5 dpf and 3 to 6 dpf. (D, E) RT-qPCR showed scpp5 expression after overexpression of scpp5 during 2 to 5 dpf and 3 to 6 dpf. (F, G) Three-dimensional reconstruction from Z-stack images of zebrafish teeth under alizarin red staining after overexpression of scpp5 during 2 to 5 dpf and 3 to 6 dpf (scale bars, 100 µm). (H, I) Antibody staining of Dendra2 after overexpression of scpp5 during 2 to 5 dpf and 3 to 6 dpf (scale bars, 50 µm). The bar graph presents the mean and standard deviation, P value *** P < .01 and **** P < .0001 were calculated by one-way ANOVA. dpf, days post-fertilization. [file mmc2.jpg]

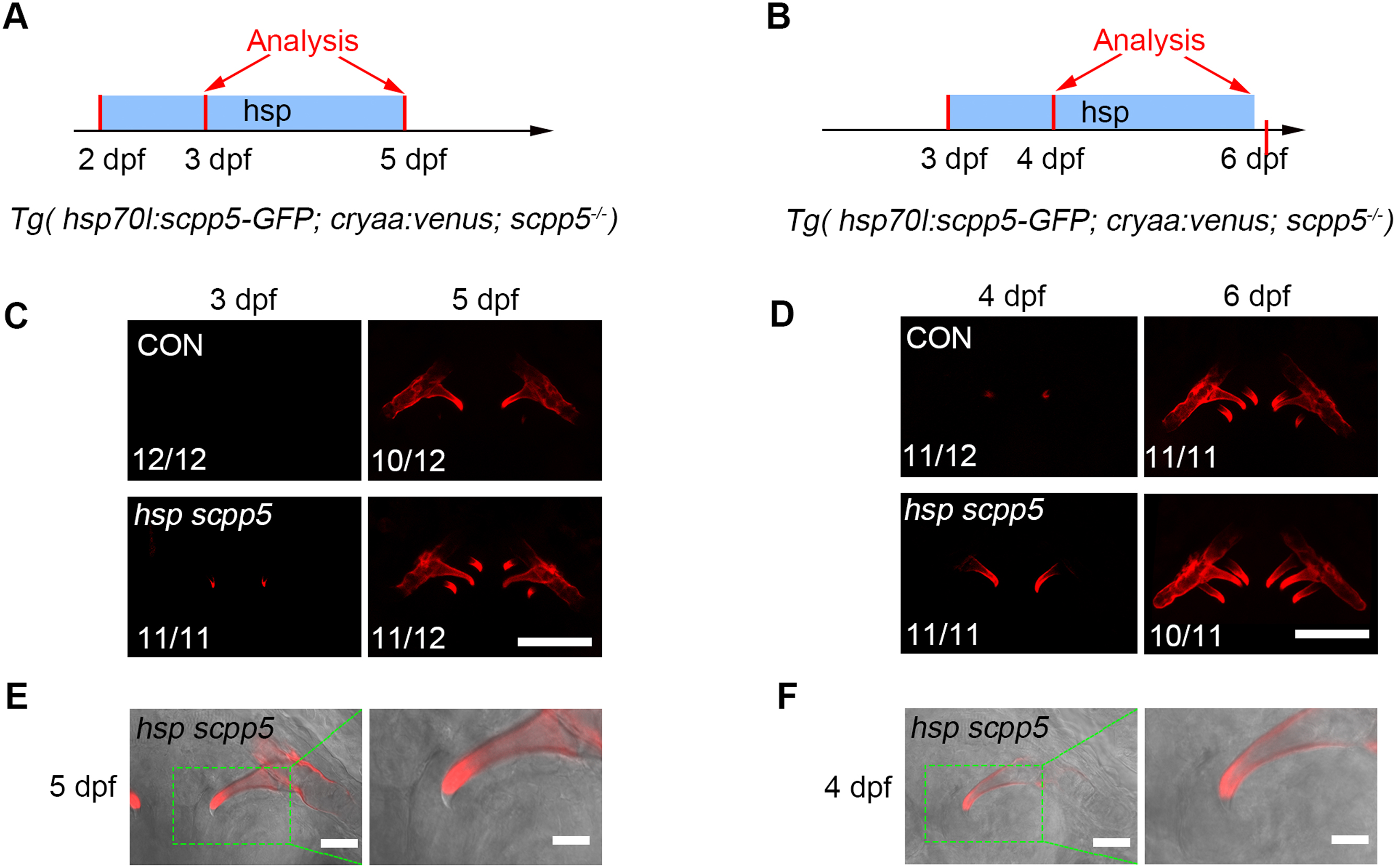

Supplement: Supplementary file 3 — Fig. S3 Restoration of tooth mineralization by scpp5 overexpression in scpp5-/- zebrafish. (A, B) Experimental schedule. Overexpression of scpp5 during 2 to 5 dpf and 3 to 6 dpf in scpp5-/- zebrafish. (C, D) Three-dimensional reconstruction from Z-stack images of zebrafish teeth under alizarin red staining after overexpression of scpp5 during 2 to 5 dpf and 3 to 6 dpf (scale bars, 100 µm). (E) Two-dimensional sectioned images of 4V1 under alizarin red staining and brightfield views in hsp scpp5 group from at 5 dpf (scale bars, left 200 µm, right 100 µm). (F) Two-dimensional sectioned images of 4V1 under alizarin red staining and brightfield views in hsp scpp5 group from at 4 dpf (scale bars, left 200 µm, right 100 µm). dpf, days post-fertilization. [file mmc3.jpg]

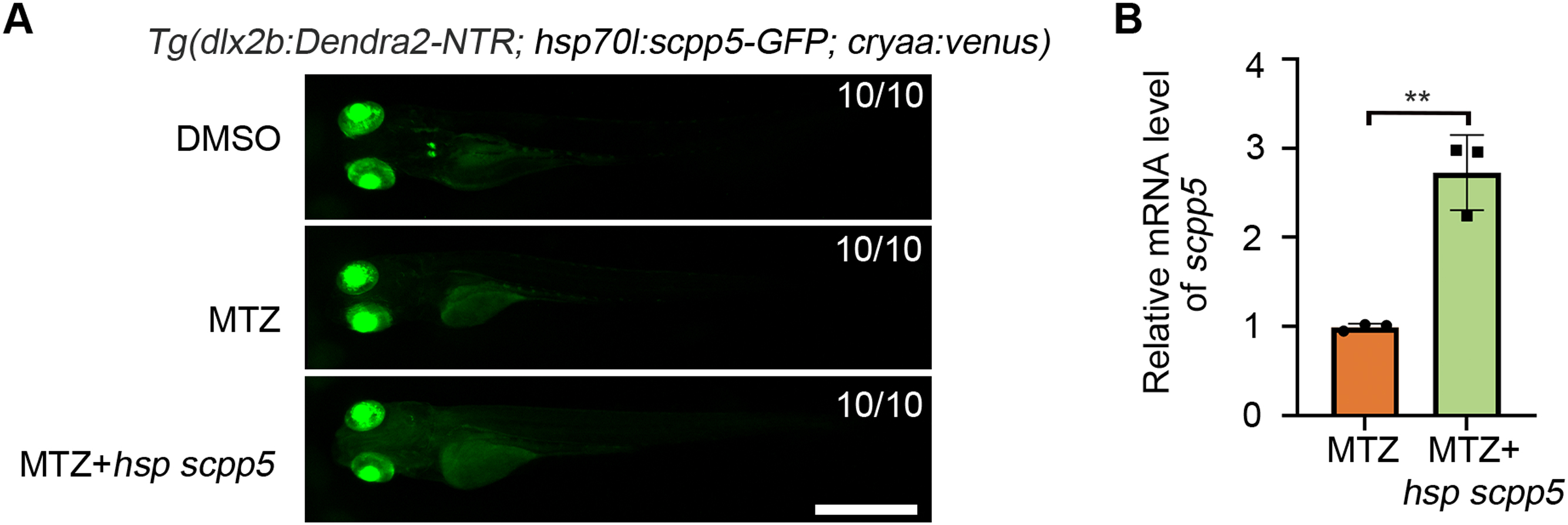

Supplement: Supplementary file 4 — Fig. S4 The effect of Tg(hsp70l:scpp5-GFP; cryaa:venus) on the expression of scpp5. (A) In vivo fluorescence images of Tg(dlx2b:Dendra2-NTR; hsp70l:scpp5-GFP; cryaa:venus) zebrafish with or without MTZ and heat-shock treatment at R0D. (B) RT-qPCR showed scpp5 expression after overexpression of scpp5 at R1D (scale bars, 1 mm). The bar graph presents the mean and standard deviation, P value ** P < .01 was calculated by t-test. MTZ metronidazole; R1D, repair 1 day. [file mmc4.jpg]

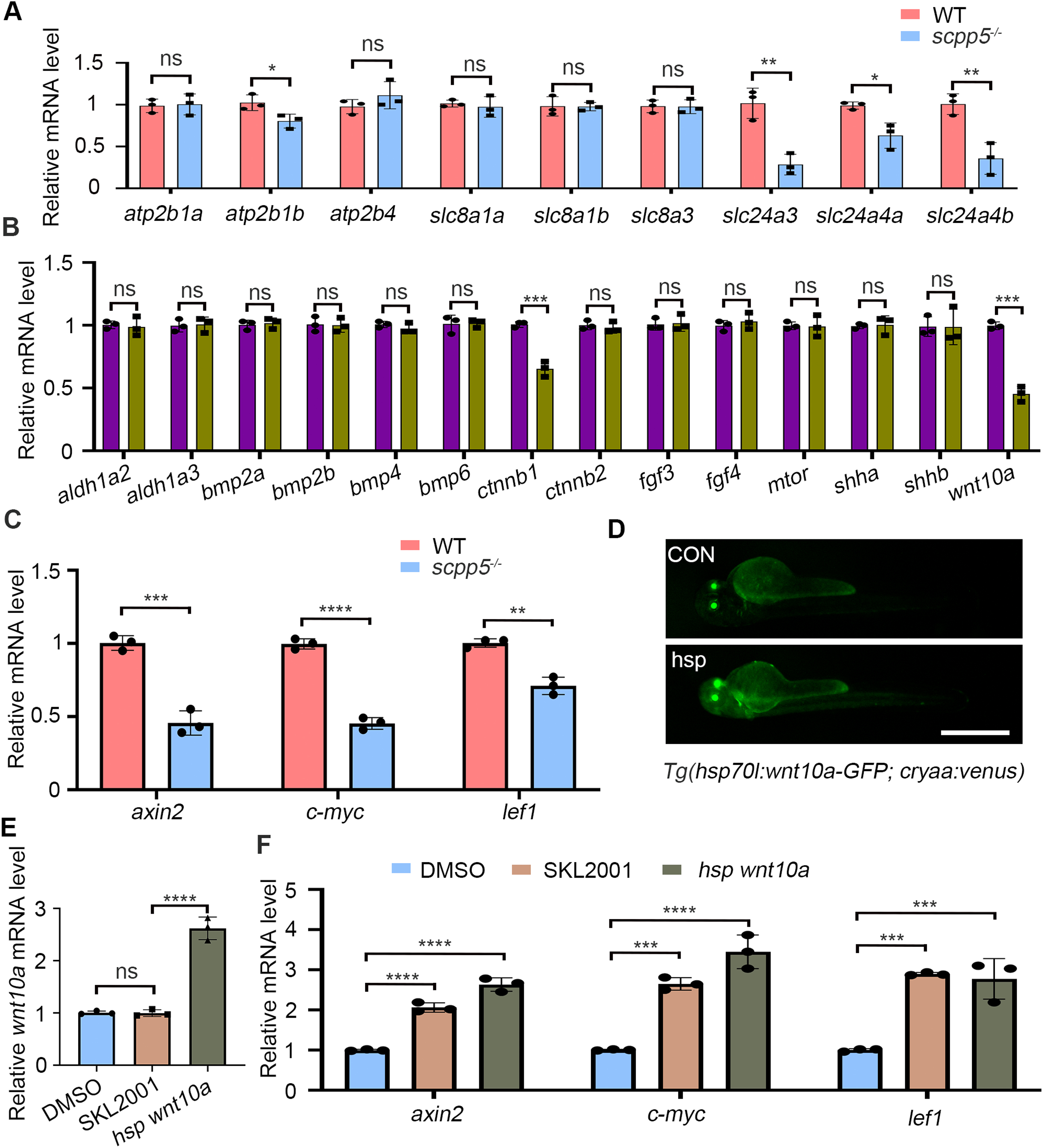

Supplement: Supplementary file 5 — Fig. S5 Effects of scpp5 knockout on gene expression and validation of Wnt/β-catenin pathway activation in zebrafish. (A) RT-qPCR showed the expression of genes related to calcium efflux channel in WT and scpp5-/- zebrafish at 4 dpf. (B) RT-qPCR showed the expression of signaling pathway genes in WT and scpp5-/- zebrafish at 4 dpf. (C) RT-qPCR showed the expression of Wnt/β-catenin signaling target genes (axin2, c-myc, and lef1) in WT and scpp5-/- zebrafish at 4 dpf. (D) In vivo fluorescence images of Tg(hsp70l:wnt10a-GFP; cryaa:venus) with or without heat-shock at 2dpf (scale bars, 1 mm). (E) RT-qPCR showed the expression of wnt10a at 4 dpf after activating Wnt-β-catenin signaling. (F) RT-qPCR showed the expression of Wnt/β-catenin signaling target genes (axin2, c-myc, and lef1) after activating Wnt/β-catenin signaling in scpp5-/- zebrafish at 4 dpf. The bar graph presents the mean and standard deviation, P value ns, not significant, * P < .05, ** P < .01, *** P < .001, and **** P < .0001 were calculated were calculated by t-test (A-C) or one-way ANOVA (E, F). dpf, days post-fertilization. [file mmc5.jpg]

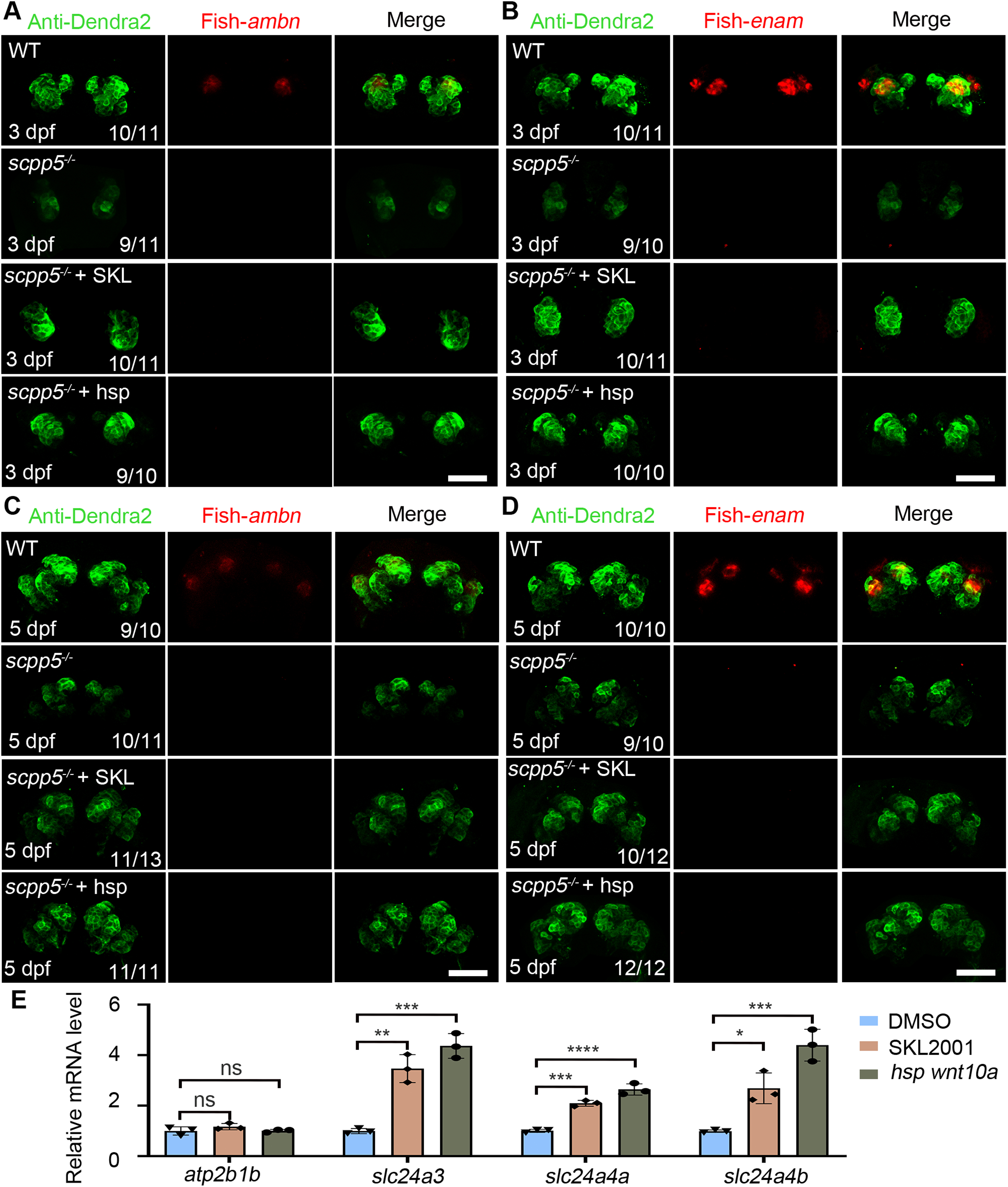

Supplement: Supplementary file 6 — Fig. S6 Effects of scpp5 knockout and Wnt/β-catenin pathway activation on enameloid matrix gene expression and calcium efflux channel genes in zebrafish. (A) FISH analysis of ambn expression in WT, scpp5-/-, and scpp5-/- zebrafish following activation of the Wnt/β-catenin signaling pathway at 3 dpf (scale bars, 50 µm). (B) FISH analysis of enam expression in WT, scpp5-/-, and scpp5-/- zebrafish following activation of the Wnt/β-catenin signaling pathway at 3 dpf (scale bars, 50 µm). (C) FISH analysis of ambn expression in WT, scpp5-/-, and scpp5-/- zebrafish following activation of the Wnt/β-catenin signaling pathway at 5 dpf (scale bars, 50 µm). (D) FISH analysis of enam expression in WT, scpp5-/-, and scpp5-/- zebrafish following activation of the Wnt/β-catenin signaling pathway at 5 dpf (scale bars, 50 µm). (E) RT-qPCR analysis showing that activation of the Wnt/β-catenin pathway modulates the expression of specific calcium efflux channel genes that are downregulated in scpp5-/- zebrafish compared to WT at 4 dpf. The bar graph presents the mean and standard deviation, p value ns, not significant, * P < .05, ** P < .01, *** P < .001, and **** P < .0001 were calculated were calculated by one-way ANOVA. dpf, days post-fertilization; hsp, hsp wnt10a; SKL, SKL2001. [file mmc6.jpg]

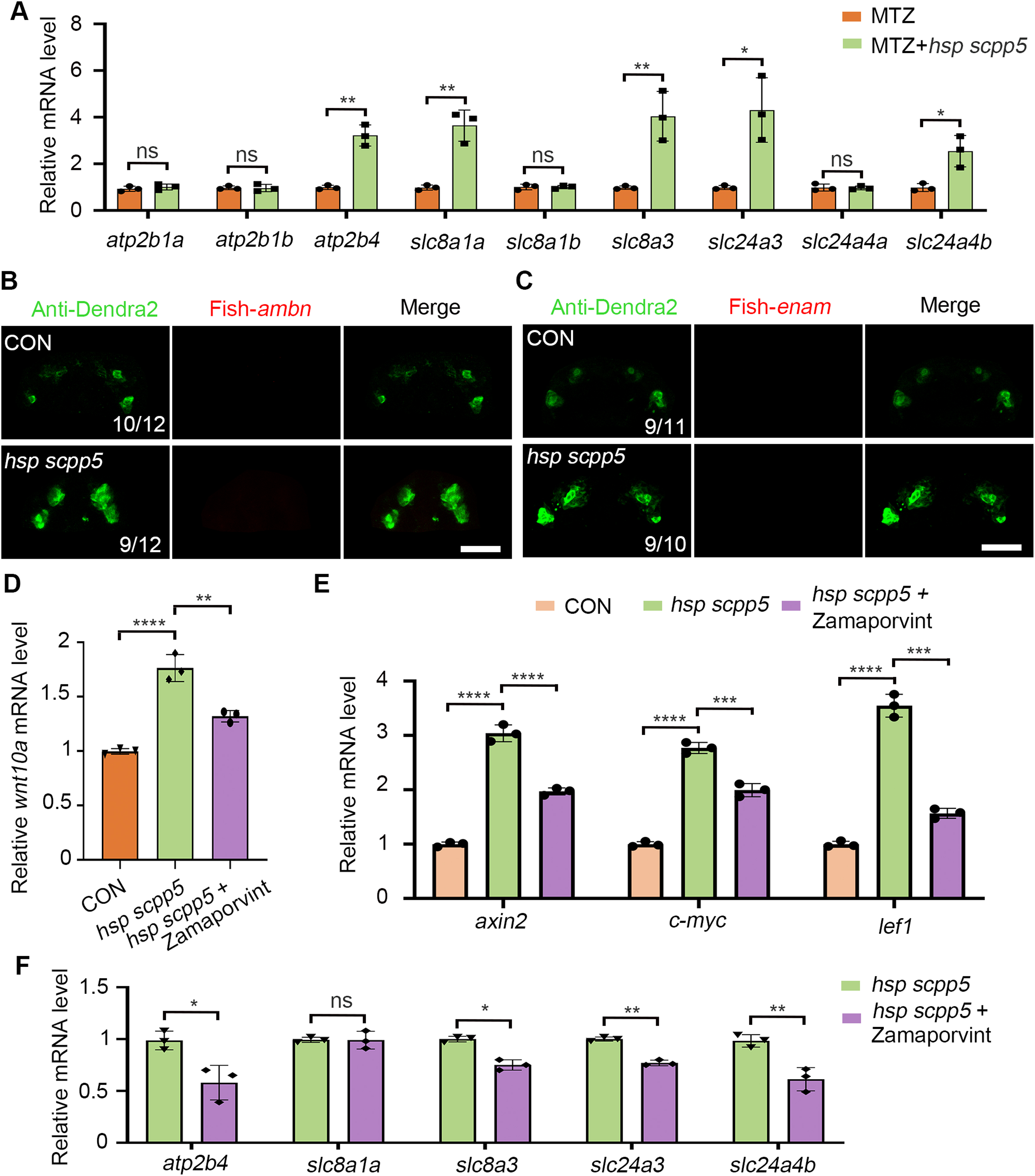

Supplement: Supplementary file 7 — Fig. S7 Effects of scpp5 overexpression on calcium efflux channel genes, enameloid matrix genes, and the Wnt/β-catenin signaling pathway at R1D, and modulation by pathway inhibition. (A) RT-qPCR analysis of calcium efflux channel-related gene expression at R1D following scpp5 overexpression. (B) FISH analysis of Dendra2 and ambn expression at R1D following scpp5 overexpression (scale bars, 50 µm). (C) FISH analysis of Dendra2 and enam expression at R1D following scpp5 overexpression (scale bars, 50 µm). (D) RT-qPCR analysis of wnt10a expression at R1D. (E) RT-qPCR analysis of Wnt/β-catenin target gene expression (axin2, c-myc, lef1) at R1D following scpp5 overexpression. (F) RT-qPCR analysis showing that Wnt/β-catenin pathway inhibition modulates the expression of specific calcium efflux channel genes that are upregulated upon scpp5 overexpression at R1D. [file mmc7.jpg]
